# CVID-Associated B Cell Activating Factor Receptor Variants Change Receptor Oligomerization, Ligand Binding, and Signaling Responses

**DOI:** 10.1007/s10875-022-01378-3

**Published:** 2022-10-29

**Authors:** Violeta Block, Eirini Sevdali, Mike Recher, Hassan Abolhassani, Lennart Hammarstrom, Cristian R. Smulski, Manuela Baronio, Alessandro Plebani, Michele Proietti, Matthaios Speletas, Klaus Warnatz, Reinhard E. Voll, Vassilios Lougaris, Pascal Schneider, Hermann Eibel

**Affiliations:** 1 Department of Rheumatology and Clinical Immunology, Medical Center and Faculty of Medicine, University of Freiburg, Freiburg, Germany; 2 Center for Chronic Immunodeficiency, Medial Center and Faculty of Medicine, University of Freiburg, Freiburg, Germany; 3 Immunodeficiency Clinic and Laboratory, Medical Outpatient Unit and Department Biomedicine, University Hospital and University of Basel, Basel, Switzerland; 4 Department of Biosciences and Nutrition, Karolinska Institutet, Huddinge, Sweden; 5 Research Center for Immunodeficiencies, Pediatrics Center of Excellence, Children’s Medical Center, Tehran University of Medical Sciences, Tehran, Iran; 6 Medical Physics Department, Centro Atómico Bariloche, Comisión Nacional de Energía Atómica (CNEA), Consejo Nacional de Investigaciones Científicas Y Técnicas (CONICET), San Carlos de Bariloche, Río Negro, Argentina; 7 Department of Clinical and Exp. Sciences, University of Brescia, Brescia, Italy; 8 Institute for Immunodeficiency, Medical Center and Faculty of Medicine, University of Freiburg, Freiburg, Germany; 9 Department of Immunology & Histocompatibility, Faculty of Medicine, University of Thessaly, Larissa, Greece; 10 Department of Biochemistry, University of Lausanne, Epalinges, Switzerland

**Keywords:** BAFFR, BAFF, CVID, PI3K, NF-kB2, SNVs

## Abstract

**Purpose:**

Binding of the B cell activating factor (BAFF) to its receptor (BAFFR) activates in mature B cells many essential pro-survival functions. Null mutations in the *BAFFR* gene result in complete BAFFR deficiency and cause a block in B cell development at the transition from immature to mature B cells leading therefore to B lymphopenia and hypogammaglobulinemia. In addition to complete BAFFR deficiency, single nucleotide variants encoding BAFFR missense mutations were found in patients suffering from common variable immunodeficiency (CVID), autoimmunity, or B cell lymphomas. As it remained unclear to which extent such variants disturb the activity of BAFFR, we performed genetic association studies and developed a cellular system that allows the unbiased analysis of BAFFR variants regarding oligomerization, signaling, and ectodomain shedding.

**Methods:**

In addition to genetic association studies, the BAFFR variants P21R, A52T, G64V, DUP92-95, P146S, and H159Y were expressed by lentiviral gene transfer in DG-75 Burkitt’s lymphoma cells and analyzed for their impacts on BAFFR function.

**Results:**

Binding of BAFF to BAFFR was affected by P21R and A52T. Spontaneous oligomerization of BAFFR was disturbed by P21R, A52T, G64V, and P146S. BAFF-dependent activation of NF-κB2 was reduced by P21R and P146S, while interactions between BAFFR and the B cell antigen receptor component CD79B and AKT phosphorylation were impaired by P21R, A52T, G64V, and DUP92-95. P21R, G64V, and DUP92-95 interfered with phosphorylation of ERK1/2, while BAFF-induced shedding of the BAFFR ectodomain was only impaired by P21R.

**Conclusion:**

Although all variants change BAFFR function and have the potential to contribute as modifiers to the development of primary antibody deficiencies, autoimmunity, and lymphoma, P21R is the only variant that was found to correlate positively with CVID.

**Supplementary Information:**

The online version contains supplementary material available at 10.1007/s10875-022-01378-3.

## Introduction

Most mature B cells require the B cell activating factor of the TNF family (BAFF) and its receptor BAFFR to survive [1]. Different from other TNF receptor family members, BAFFR has a single extracellular cysteine-rich domain (CRD) [2] that is needed for the pre-assembly into multimers as well as for ligand binding [3]. When BAFF binds to BAFFR, it activates the non-canonical nuclear factor kappa B (NF-κB2) pathway [4, 5], which is a slow process as it requires the de novo synthesis and accumulation of NIK [6]. NF-κB2 activity then upregulates anti-apoptotic genes that support the survival of peripheral B cells [7–10]. BAFFR also activates the phosphoinositide-3 kinase (PI3K) and extracellular signal regulated kinases (ERK), which coordinately control B cell survival, metabolic responses, and cellular fitness [8, 9, 11–15].

In mice, the inactivation of *Baffr* [16] or *Baff* [17] blocks B cell development beyond the stage of transitional B cells reducing drastically the number of follicular and marginal zone B cells. In humans, complete BAFFR deficiency has been found by screening patients with common variable immunodeficiency (CVID). The homozygous deletion removed part of the BAFFR transmembrane (TM) region and resulted in severe B lymphopenia, the loss of marginal zone and switched memory B cells, low serum immunoglobulin M (IgM) and IgG levels, and strongly impaired T cell-independent B cell responses [18].

In addition to this deletion, several single nucleotide variants (SNVs) in the *BAFFR* gene (*TNFSRF13C*) have been reported in CVID patients [3, 19–21]. A guanine to cytosine exchange in exon 1 (c.62C > G, rs77874543) results in the amino acid substitution of proline21 by arginine (P21R) and occurs with an allele frequency in the general population of approximately 8%. Pro21 is completely conserved in primates and resides in a loop of CDR1 close to the BAFF binding site that forms the pre-ligand assembly domain (PLAD) of BAFFR. Therefore, the change of Pro21 to Arg21 disturbs ligand-independent and ligand-enhanced multimerization of BAFFR [3].

The guanine-191-to-thymine (c.191G > T, rs547352394) exchange has been described first in CVID [19]. It has an allele frequency of 1.5% and substitutes within a highly conserved region of the extracellular domain glycine for valine at position 64 (G64V).

The rs151243201 (c.43C > T, p.P146S) variant is encoded by exon 3. It has a highly variable allele frequency of 1/395 in African/African American population compared to < 1/128,530 alleles in European non-Finnish population and affects an evolutionary well-conserved proline-rich motif (PxWPP) in the intracellular domain.

The H159Y variant (c.475C > T, rs1756766) changes His159 against Tyr affecting a region that is conserved between birds and mammals, as it is directly adjacent to the TNF receptor-associated factor (TRAF3) recognition motif PVPAT. The H159Y variant results in a hyperactive receptor with enhanced recruitment of TRAF3 and TRAF6 [20, 22]. H159Y has an increased prevalence in patients suffering from Sjögren’s syndrome-related lymphoma, non-Hodgkin lymphoma, and multiple sclerosis [20, 22, 23]. H159Y and P21R were found to co-segregate [21, 23] suggesting that this allelic combination had a selective advantage.

Apart from these known BAFFR variants, we evaluate here two novel BAFFR variants detected in individuals with CVID. The first variant that been discovered in a CVID patient results from a guanine-154-to-adenine (c.154G > A) exchange substituting a highly conserved alanine with threonine at position 52 (A52T). The rs776259962 SNP is an extremely rare allele and results in the duplication of four evolutionary conserved amino acids (LALV) ranging from position 92 to 95 in the TM region (DUP92-95).

Thus far, it remained unclear to which extent these BAFFR variants affect B cell survival and if they could contribute to disease development. To gain further insight into the causal relation between the BAFFR variants and their impact on BAFFR function and activity, we expressed these variants in the human Burkitt’s lymphoma cell line DG-75 and analyzed BAFFR oligomerization, BAFFR processing, and the activation of downstream signaling pathways. Our findings reveal that the variants affect functional properties of BAFFR and may contribute as modifying factors to an increased risk of developing primary immunodeficiency, autoimmunity, or B lymphomagenesis.

## Methods

All methods and material are described in the supplemental information.

## Results

### Association of BAFFR Variants with CVID

**Fig. 1:**
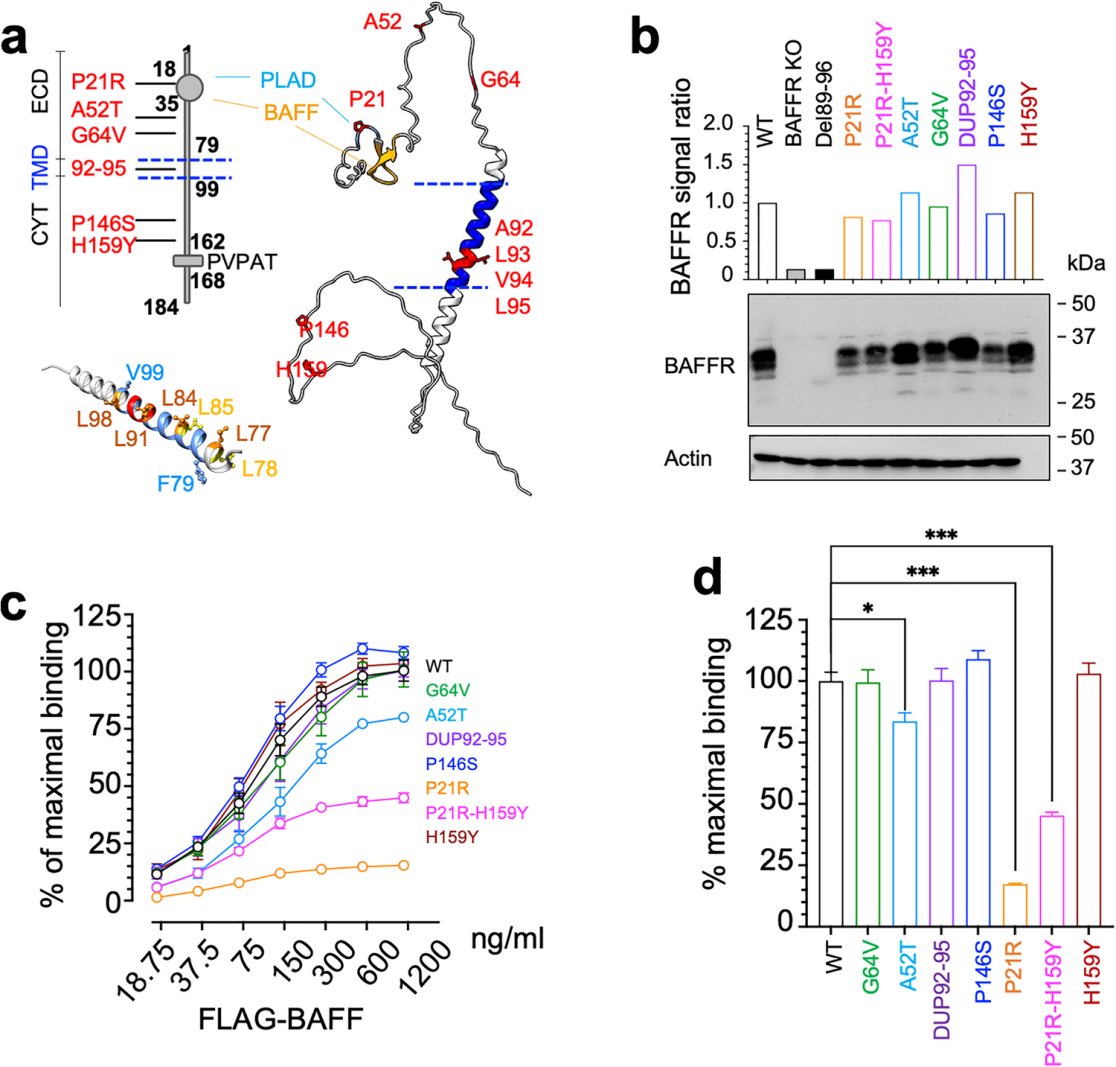
Expression of BAFFR variants. **a** Schematic representation of BAFFR structure showing the localization of the variants. ECD, extracellular domain; TMD, transmembrane region; CYT, cytoplasmic domain; PLAD, pre-ligand assembly domain; PVPAT, TRAF3 binding motif. The 3D ribbon model of the BAFFR polypeptide chain is based on the entry Q96RJ3 of Alphafold [49]. The residues changed by the variants are shown in red, the BAFF binding site in orange, the PLAD in light blue, and the TM region in blue. The helical structure of the TM region ranging from F79 to V99 has the potential of forming two different leucine zippers (L77, L84, L91, and L98 (orange) and L78 and L85 (yellow)). The residues affected by the duplication 92–95 are shown in red. **b** Western blot analysis of BAFFR expression in DG-75 BAFFR KO cells transduced with the different BAFFR variants expressed from lentiviral vectors carrying GFP separated from BAFFR by an IRES. After densitometric scanning of western blots, BAFFR signals were normalized to the corresponding actin signals and the signal ratio was calculated as (BAFFR signal [variant])/(BAFFR signal [WT]). **c **Dose-dependent binding of FLAG-tagged BAFF to WT BAFFR and to the variants expressed as GFP fusion proteins by DG-75 cells. FLAG-tagged BAFF was detected by flow cytometry using anti FLAG-tagged APC-conjugated antibodies. MFI of BAFF bound to the surface of cells was normalized to BAFFR expression (GFP), and the percentage of bound BAFF was calculated as (BAFFR variant signal [n ng/ml])/(WT BAFFR signal [1200 ng/ml]) × 100. Signals recorded from cells incubated only with the anti-FLAG-tagged antibody (0 ng/ml BAFF) were used to determine the background signal intensities. Data show means and SD of four independent experiments. **d **BAFF binding capacity of BAFFR variants relative to WT BAFF determined as (BAFFR variant signal [1200 ng/ml])/(WT BAFFR signal [1200 ng/ml]) (**c**). Significant differences were calculated by Brown-Forsythe and Welch ANOVA. *P* ≥ 0.1234 (ns); *P* ≤ 0.0032 (*); *P* ≤ 0.0021 (**); *P* ≤ 0.0002 (***); *P* < 0.00001 (****)

The association of the BAFFR variants (Fig. 1a, Supplemental Table S1) with CVID was determined from our whole exome sequencing data of our CVID patients and controls and compared to the allele frequencies retrieved from the Genome Aggregation Database gnomAD [24] (Table 1). B cell populations of patients carrying these variants are shown in Supplemental Figure S1. In line with our previous data [3], P21R was found to correlate with CVID (*P* = 0.0458). The FACS profiles in Figure S1 are from a heterozygous CVID patient with low percentage of switched memory B cells.

**Table 1:** Allele frequencies of BAFFR variants

Cohort	Variant	rsID	Ref	Alternate	No of variant alleles	No. of WT alleles	Allele frequency (%)	*P* value	RR	OR
CVID*	G64V	rs547352394	C	A	18	1064	1.64			
Control**					6	656	0.91	0.229	1.785	1.798
gnomAD***					737	50,481	1.46	0.488	1.140	1.143
CVID	DUP92-95	rs776259962	A	ACCAGGACCAGCG	1	608	0.164			
Control					NA	NA				
gnomAD					1	1,045,517	0.00096	0.0116	171.6	171.9
CVID	P146S	rs151243201	G	A	0	331	NA			
Control					NA	NA	NA			
gnomAD					0	128,530	NA			
CVID	P21R	rs77874543	G	C	100	880	11.36			
Control					166	1922	8.64	0.0458	1.284	1.316
gnomAD					3213	37,059	8.67	0.0145	1.279	1.311
CVID	H159Y	rs61756766	G	A	5	663	1.28			
Control					11	1133	0.97	0.533	1.319	1.323
gnomAD					961	127,323	0.75	0.825	1.706	1.715
CVID	P21R-H159Y				12	795	1.51			
Control					8	622	1.29	0.8233	1.171	1.174
gnomAD					NA	NA	NA			

Allele counts are from the cohorts of CVID patients (*) and healthy donors (controls, **) established and analyzed at the participating institutions. ***The allele counts, the rsIDs, the reference, and the alternate alleles are derived from the European (non-Finnish) population of the Genome Aggregation Database (gnomAD; https://gnomad.broadinstitute.org) published in Karczewski K.J., Francioli L.C., and Tiao G. et al. (2020). The mutational constraint spectrum quantified from variation in 141,456 humans. Nature 581, 434–443. *P* values were calculated applying a two-sided Fisher’s exact *t*-test relative to their presence in our control cohort and relative to the gnomAD cohort. The allele counts of P21R-H159Y double mutant in the gnomAD cohort are not known. Alleles encoding the P146S and DUP92-95 variants were not detected in our control cohorts. RR, relative risk; CVID vs. control, and CVID vs. gnomAD; OR, odds ratio; CVID vs. control, and CVID vs. gnomAD; NA, data not available

H159Y encoding alleles were detected at a slightly higher frequency in the CVID cohort (1.28%) than in the control (0.97%) or in the gnomAD cohort of the European, non-Finnish population (0.75%). The FACS profiles displayed in Figure S1 show B cells from a homozygous patient.

The patient carrying G64V is heterozygous for the variant allele, and the encoding rs547352394 SNP is slightly but not significantly overrepresented in the CVID cohort (1.64%) compared to the controls (0.91%) and to the gnomAD cohort (1.46%).

The DUP92-95 variant (rs776259962) was found only once in the CVID cohort. Inherited from a clinical asymptomatic mother, the patient carried the mutation in a heterozygous form. This variant is extremely rare as it is reported only once in the gnomAD European non-Finnish population (1/104,518 alleles).

The A52T variant is heterozygous and was detected so far only in one CVID patient but neither in our control nor in the gnomAD cohorts.

P146S has not been found yet in CVID patients. The SNP rs151243201 occurs in 1/395 alleles of the African/African American population, but it has not yet been found in 128,530 alleles of the European non-Finnish population.

Except for the patient shown in Supplemental Figure S1a, the H159Y variant was detected so far only in persons who also carry the P21R variant. Since the expected frequency for the random association of P21R and H159Y is 0.08–0.09%, while the actual frequency of P21R combined with H159Y is in the range of 1.5% (CVID) to 1.3% (controls), the P21R-H159Y alleles are in a linkage disequilibrium with a coincidence that is 10–15 times higher than expected. DNA sequence analysis of one P21R-H159Y allele revealed that the SNPs are encoded by the same strand of DNA.

### BAFFR Expression and Ligand Binding Activities of the Different BAFFR Variants

Phenotypic analysis of B cells from CVID patients carrying the different BAFFR variants (Supplemental Figure S1) revealed only small populations of IgD^−^ CD27^+^ switched memory cells, while the percentages of naïve (IgD^+^ CD27^−^) and marginal zone (MZ) B cells were in the normal range [25, 26], except for the A52T patient, who had recovered from treatment with the anti-CD20 mAb rituximab to control autoimmune cytopenia (Supplementary Fig. S1a,b). Compared to the matched controls, BAFFR surface expression was lower on B cells of the P21R, G64V, and DUP92-95 carriers and slightly higher for the individual with H159Y, indicating that the variants could affect BAFFR expression directly or indirectly.

**Fig. 2:**
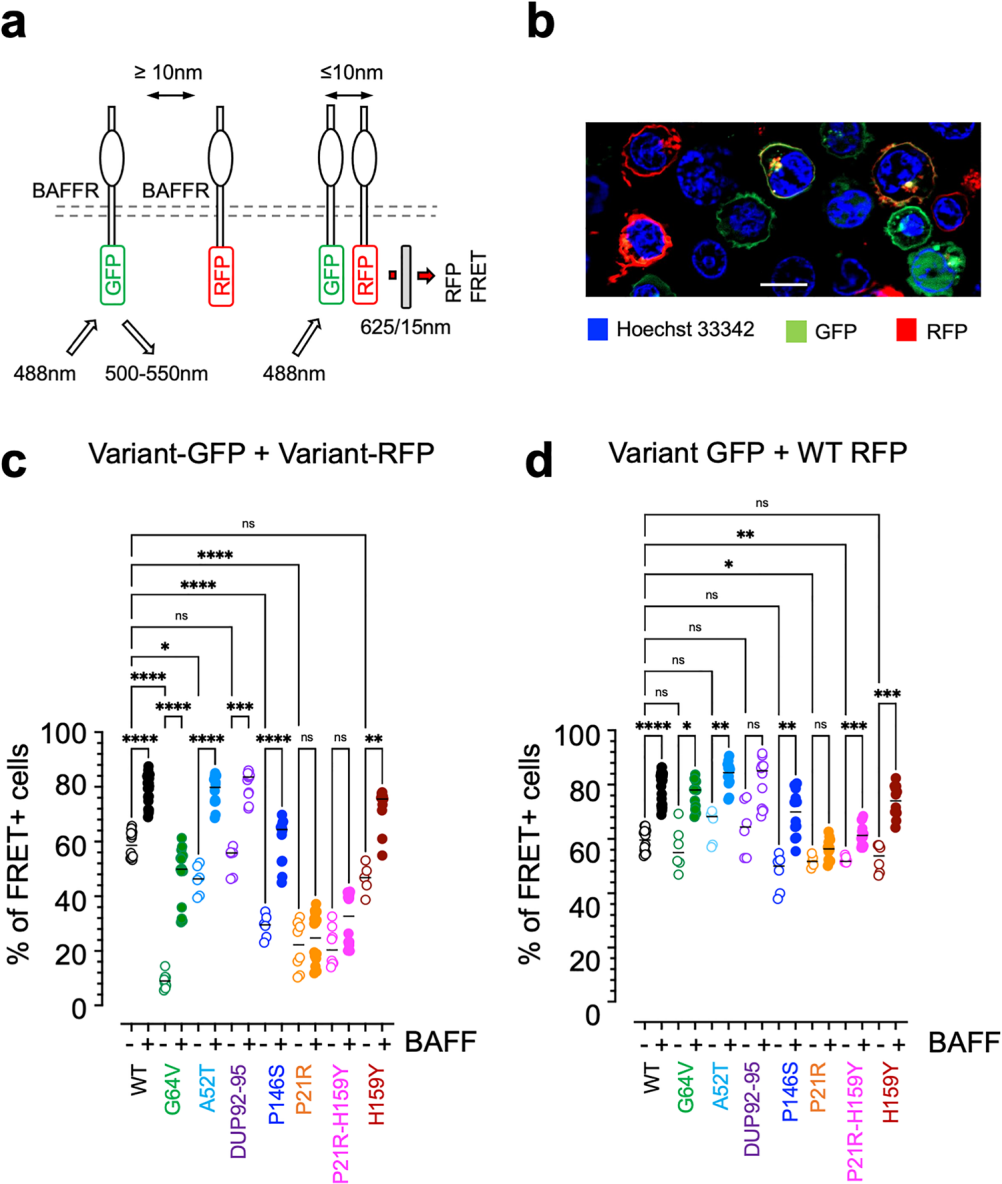
BAFFR variants change oligomerization. **a** Schematic representation of FRET analysis performed with DG-75 BAFFR KO cells expressing BAFFR-GFP and BAFFR-RFP fusion proteins. Excitation of GFP by the 488 nm laser results in the emission of green light of 500–550 nm wavelength. If the distance between the donor (GFP) and acceptor (RFP) fluorophore is ≤ 10 nm, the excitation of BAFFR-GFP allows radiation-free energy transfer (FRET) to BAFFR-RFP resulting in the excitation of RFP and the emission or red light passing a 625/15-nm bandpass filter. **b** Fluorescence of DG-75 BAFFR KO cells co-transduced with BAFFR-WT-GFP (green) and BAFFR-WT-RFP (red) fusion proteins. Nuclei were stained with Hoechst 33342 (blue). Scale bar: 10 µm. **c** Percentage of FRET positive DG-75 BAFFR KO cells co-expressing WT-BAFFR or variant GFP- and RFP-fusion proteins. Open symbols represent FRET of unstimulated cells, filled symbols FRET of cells stimulated with 50 ng/ml BAFF for 1 h at 37 °C. Each data point corresponds to an independent replicate. Differences between the WT BAFFR and the BAFFR variants were calculated by Brown-Forsythe and Welch ANOVA. *P* ≥ 0.1234 (ns); *P* ≤ 0.0032 (*); *P* ≤ 0.0021 (**); *P* ≤ 0.0002 (***); *P* < 0.00001 (****). The detailed gating strategy and the increase of FRET signal intensity after adding BAFF is shown in Supplemental Figure S3. **d** FRET analysis of DG-75 BAFFR KO cells co-expressing WT BAFFR-RFP and WT or variant BAFFR-GFP fusion proteins. Open symbols represent untreated cells, filled symbols cells stimulated with 50 ng/ml BAFF for 1 h at 37 °C. Each data point represents an independent replicate. Differences between the WT BAFFR and the BAFFR variants were calculated by Brown-Forsythe and Welch ANOVA

**Fig. 3:**
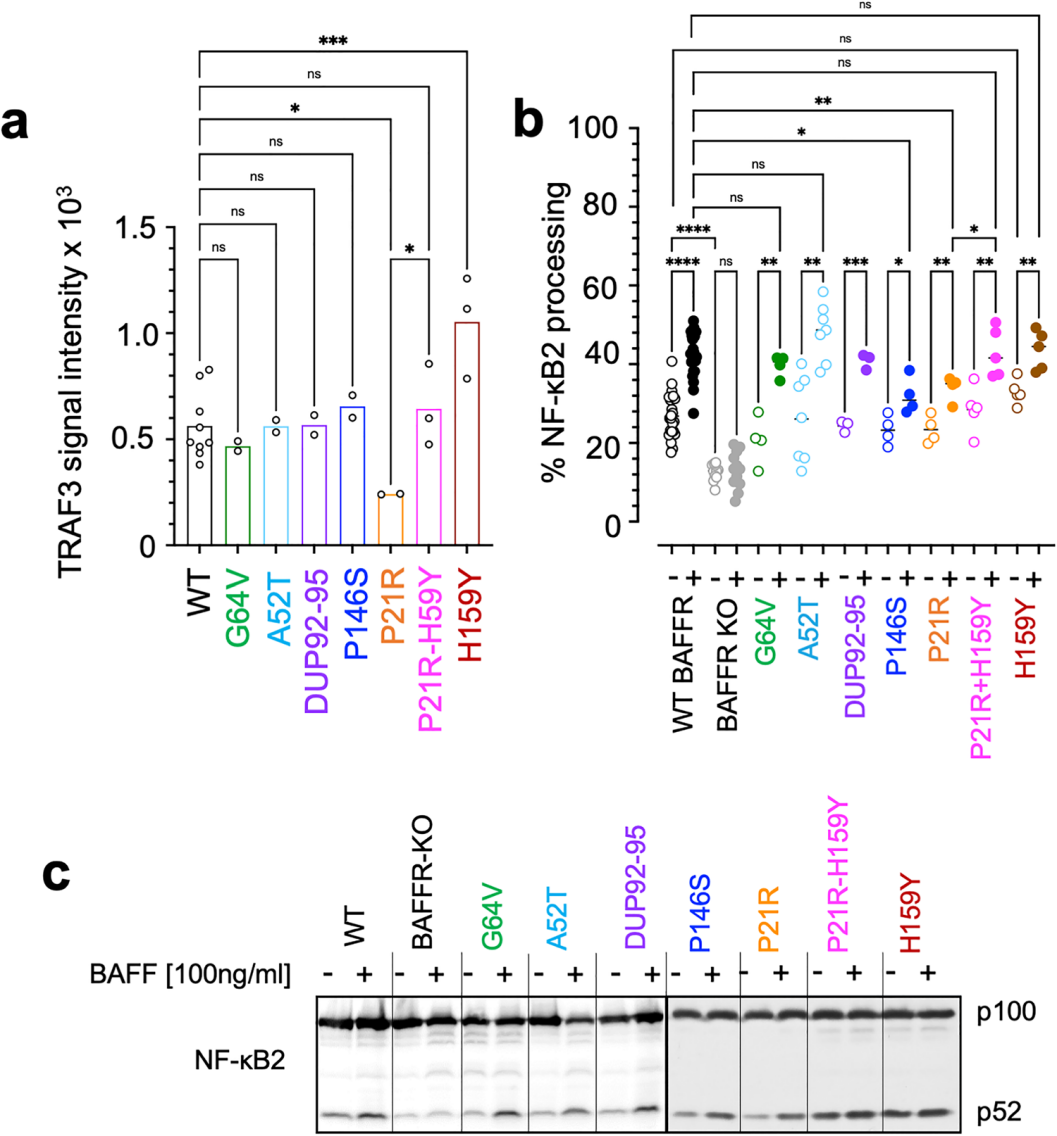
The P21R variant impairs activation of NF-κB2. **a** TRAF3 recruitment by WT and variant BAFFR. The signal intensities of TRAF3 were determined by densitometric scanning of western blots of TRAF3 co-immunoprecipitating with BAFFR and FLAG-tagged BAFF. DG-75 BAFFR KO cells expressing WT or variant BAFFRs were incubated with FLAG-tagged BAFF for 60 min at 37 °C. FLAG-tagged BAFF bound BAFFR was then immunoprecipitated using anti-FLAG-M2 IgG coupled to magnetic beads as shown in Supplemental Figure S4. To account for differences in cell numbers and protein amounts between different samples, the signal intensities of co-immunoprecipitated TRAF3 were normalized to the intensities of TRAF3 signals determined by western blotting of the corresponding input samples. Data points represent independent replicates. Significant differences were calculated applying Brown-Forsythe and Welch ANOVA. *P* ≥ 0.1234 (ns); *P* ≤ 0.0032 (*); *P* ≤ 0.0021 (**); *P* ≤ 0.0002 (***); *P* < 0.00001 (****). **b** BAFF-induced processing of NF-кB2. Cells were treated with 100 ng/ml BAFF over night or left untreated. BAFF-induced activation of NF-кB2 was determined by western blotting of lysates of DG-75 BAFFR KO cells expressing either WT or variant BAFFR as shown in (**c**). Signal intensities were determined by densitometric scanning of western blots. The percentages of processed NF-κB2 (p52) was calculated as (signal [p52])/(signal [p52] + signal [p100]) × 100. Each data point represents one independent experiment quantified by densitometry and significant differences between unstimulated and stimulated cells and between WT BAFFR and the BAFFR variants were calculated by Brown-Forsythe and Welch ANOVA; *P* ≥ 0.1234 (ns); *P* ≤ 0.0032 (*); *P* ≤ 0.0021 (**); *P* ≤ 0.0002 (***); *P* < 0.00001 (****). **c** Western blot analysis of NF-кB2 processing. DG-75 BAFFR KO cells were transduced with lentiviral vectors expressing WT BAFFR or BAFFR variants and GFP as marker separated from BAFFR cDNA by an IRES. GFP was used to sort transduced cells. 10^6^ cells/sample were stimulated overnight with 100 ng/ml BAFF or left untreated. After 24 h, cells were lysed and analyzed for the processing of the p100 into the p52 subunit by western blotting. The figure shown one representative experiment of at least 3 independent replicates analyzed from each BAFFR variant

**Fig. 4:**
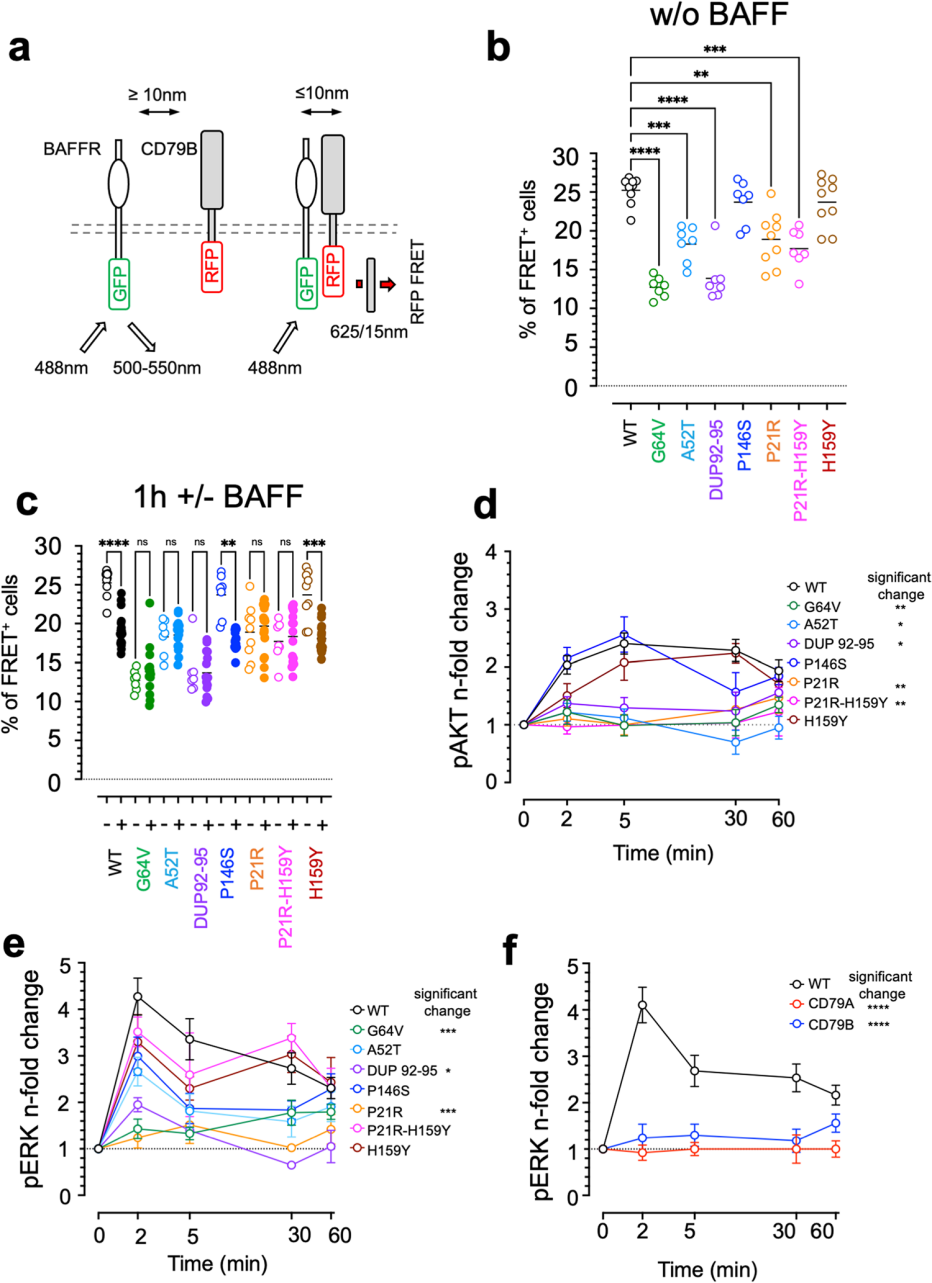
BAFFR variants impair interactions between BAFFR and the BCR component CD79B. **a** Schematic representation of FRET analysis performed with DG-75 BAFFR KO cells expressing WT or variant BAFFR-GFP and CD79B-RFP fusion proteins. If the distance between the donor (GFP) and acceptor (RFP) fluorophore is ≤ 10 nm, the excitation of BAFFR-GFP by the 488-nm laser allows FRET to CD79B-RFP. The excitation of RFP is detected by the emission or red light passing a 625/15-nm bandpass filter. **b** Percentage of FRET^+^ cells detected in cells co-expressing BAFFR-GFP and CD79B-RFP fusion proteins. Each data point corresponds to an independent replicate. **c** Percentage of FRET + cells detected before (open symbols) and 1 h after adding 100 ng/ml BAFF. The gating strategy to identify FRET ^+ ^cells and the spillover of GFP and RFP signals into the FRET-detecting channel is shown in Supplemental Figure S5. Each data point represents one independent replicate. Significant differences between WT BAFFR and the BAFFR variants and between unstimulated and stimulated cells were calculated by Brown-Forsythe and Welch ANOVA; *P* ≥ 0.1234 (ns); *P* ≤ 0.0032 (*); *P* ≤ 0.0021 (**); *P* ≤ 0.0002 (***); *P* < 0.00001 (****). **d** BAFF-induced activation of AKT. DG-75 cells expressing WT BAFFR or the different BAFFR variants from lentiviral vectors equipped with IRES-GFP to identify and sort transduced cells were treated with 100 ng/ml BAFF for the indicated time points. Whole cell lysates were analyzed for pAKT S473 by western blotting as shown for representative samples in Supplemental Figure S6. Signal intensities of pAKT S473 of each time point were determined by densitometric scanning, normalized to the actin signals of the corresponding time point (Supplemental Figure S6), and used for each sample to calculate n-fold changes relative to *t* = 0. The time course experiment represents the compilation of at least 3 different replicates/sample. Significant differences at *t* = 5 min were calculated by Brown-Forsythe and Welch ANOVA. **e** BAFF-induced phosphorylation of pERK1/2. DG-75 BAFFR KO cell lines expressing WT or variant BAFFR and GFP from an IRES to identify and sort transduced cells were activated with BAFF for different time points as indicated. The phosphorylation of ERK1/2 T202/Y204 was analyzed by western blotting as shown for the samples in Supplemental Figure S8 representing at least 1 out of 3 independent replicates per time point. After densitometric scanning of the western blots, signals were normalized to the respective actin signals detected by stripping and re-probing the blots. Using the normalized signals, *n*-fold changes relative to *t* = 0 were calculated as (signal[t = n])/(signal[t = 0]). Significant differences at *t* = 2 min were calculated by Brown-Forsythe and Welch ANOVA. **f** Inactivation of CD79A and CD79B prevents BAFF-induced ERK1/2 T202/Y204 phosphorylation. CD79A and CD79B were inactivated by CRISPR/Cas9-directed mutagenesis in DG-75 BAFFR-KO cells transduced with a lentiviral vector carrying WT BAFFR and IRES-GFP (Supplementary Figure S4b). Cells were analyzed in time-course experiments of 3 independent replicates for BAFF-induced ERK1/2 phosphorylation by western blotting as described in (**e**). The graphs shown the mean and SEM of at least 3 independent experiments. Significant differences at *t* = 2 min were calculated by Brown-Forsythe and Welch ANOVA

**Fig. 5:**
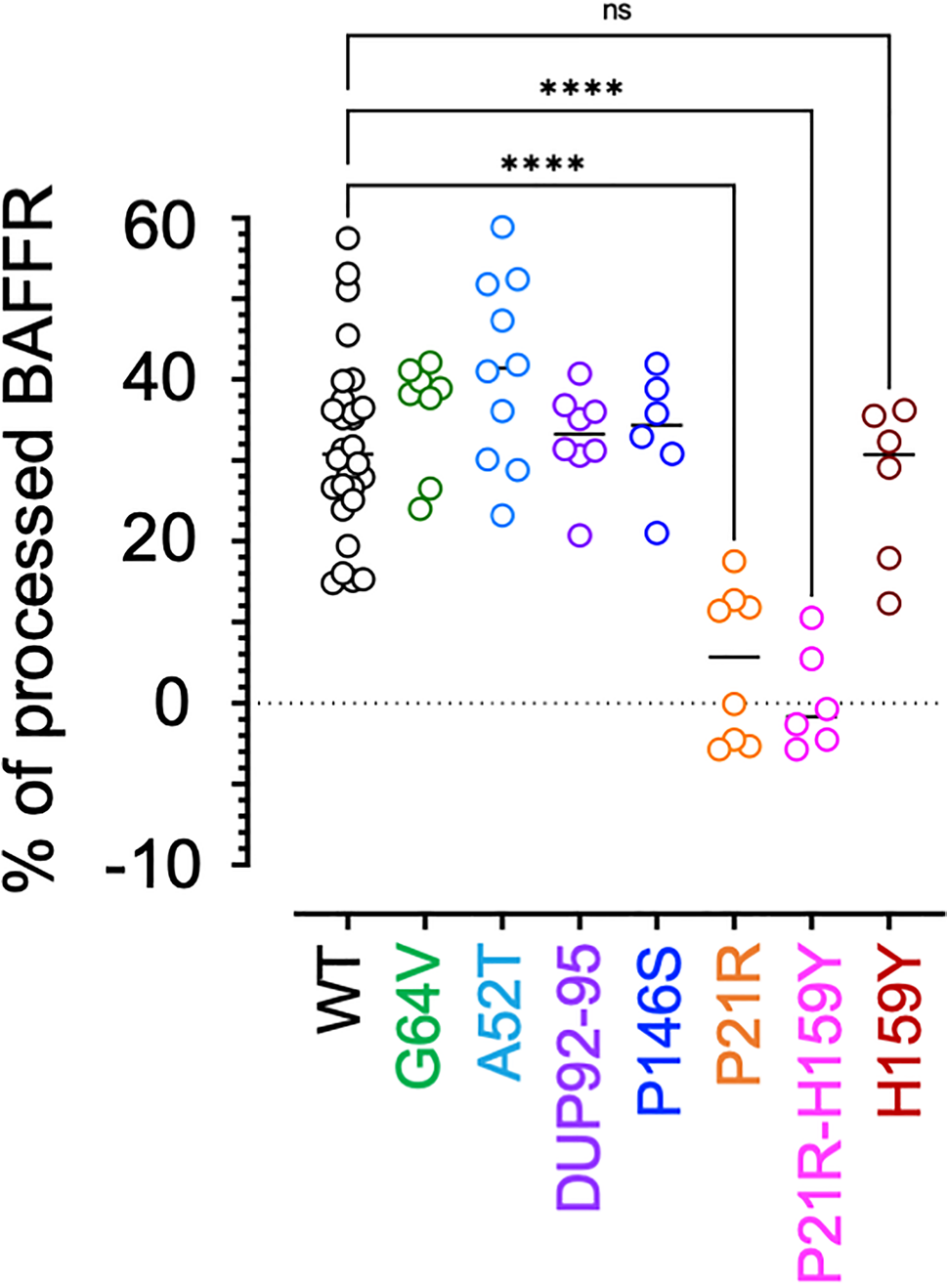
P21R impairs BAFFR processing. DG75-BAFFR KO cells expressing WT or variant BAFFR and GFP from an IRES to identify and sort transduced cells were activated over night with 100 ng/ml BAFF. Whole cell lysates were analyzed by western blotting to detect BAFFR and actin as shown by representative examples in Supplemental Figures S6, S8. After densitometric scanning, BAFFR signal intensities of the different samples were normalized to the corresponding actin signals, and the percentage of processed BAFFR was calculated as [100 – (100 × signal [100 ng/ml BAFF])/(signal[0 ng/ml])]. Each data point shows an independent replicate. Significant differences in BAFFR processing between WT BAFFR and the BAFFR variants were calculated by Brown-Forsythe and Welch ANOVA; *P* ≥ 0.1234 (ns); *P* ≤ 0.0032 (*); *P* ≤ 0.0021 (**); *P* ≤ 0.0002 (***); *P* < 0.00001 (****)

To determine if the different BAFFR variants would affect BAFFR surface expression, oligomerization, signaling, and shedding in a comparable and controlled environment, we expressed the variants by lentiviral gene transfer in the Burkitt’s lymphoma line DG-75 (Fig. S2), because this cell line allows the analysis of BAFFR-dependent activation of NF-kB2, AKT, and ERK1/2. To avoid interference with endogenously expressed BAFFR, we had inactivated the BAFFR alleles before the lentiviral transductions by CRISPR/Cas9 mutagenesis resulting in DG-75 BAFFR KO cells. In these cells, the variants were expressed either as GFP fusion proteins (Figs. 1, 2, and 4a,b) or 5′ to GFP cDNA linked by an IRES (Figs. 1, 3, 4d-f, and 5).

Western blot analysis from DG-75 cells carrying the different BAFFR-IRES-GFP constructs revealed that expression of the variants was in the range of 0.75–1.5 × of WT BAFFR levels (Fig. 1b). Flow cytometric analysis of their surface levels expressed as BAFFR-GFP fusion proteins showed varying signals depending on the monoclonal antibodies used to detect BAFFR (Supplemental Figure S2), while the expression levels of the C-terminally fused GFP ranged between 0.5 and 1.5 × of those of WT BAFFR-GFP. Thus, none of the variants prevented BAFFR expression (Supplemental Figure S2).

We then compared the ligand-binding capacity of the BAFFR variants to WT BAFFR in a dose-dependent binding assay using FLAG-tagged BAFF as ligand and anti-FLAG antibodies to detect BAFF bound to surface BAFFR. As published before [3], the BAFF-binding capacity of P21R was reduced by 83% compared to WT BAFFR (Fig. 1c, d). Reaching 84% of the BAFF-binding capacity of WT BAFFR, the A52T variant interfered with BAFF binding too (Fig. 1c, d), and because of the exchange of Pro21 against Arg, the P21R-H159Y double mutant had its ligand binding reduced by approximately 65%.

### BAFFR Variants Interfere with BAFFR Oligomerization

BAFFR assembles spontaneously into oligomers [3]; we analyzed if the different the variants would change ligand-independent and ligand-induced oligomerization by FRET with BAFFR fusion proteins linked at their C-terminal ends to green (GFP) or red fluorescent protein (RFP) (Fig. 2a). Lentiviral gene transfer of the fusion protein constructs into DG-75 BAFFR KO cells resulted in a population of GFP or RFP single positive, GFP and RFP double-positive and double negative cells (Fig. 2b, Supplemental Figure S3a,b). Interactions between BAFFR-GFP and BAFFR-RFP fusion proteins were then analyzed by flow cytometry recording the Förster resonance energy transfer (FRET) from GFP to RFP (Fig. 2a). Applying the gating strategy shown in Supplementary Figure S3, the percentage of FRET^+^ cells in the double positive population co-expressing BAFFR-GFP and BAFFR-RFP was used as a read-out system for direct interactions between the fusion proteins. Cells expressing only BAFFR-GFP or BAFFR-RFP were used to set gates for GFP to RFP FRET-positive cells.

FRET from GFP to RFP fusion proteins was then analyzed in resting cells and compared to cells that were activated for 1 h with BAFF. This approach allowed to determine if BAFFR variants would interfere with the spontaneous, ligand-independent oligomerization of BAFFR, or with BAFF-enhanced clustering of BAFFR oligomers that are cross-linked by BAFF, as it can interact via its FLAP region with other BAFF molecules allowing the formation of large BAFFR clusters [27].

Spontaneous oligomerization of BAFFR was impaired by P21R, G64V, A52T, and P146S (Fig. 2c). P21R resides in the PLAD and was found before to interfere with BAFFR oligomerization [3]. A52T and G64V locate a loop-like structure that extends into the extracellular space and connects the N-terminal CRD to the TM region (Fig. 1a). Different from these extracellular variants, DUP92-95 changes the TM region, Pro146 is the first of three conserved Pro residues in the cytoplasmic tail, and H159Y is at the TRAF3 binding site. Binding of BAFF to BAFFR immediately increased FRET-induced fluorescence (Supplemental Figure S3b-d), and this increase was observed with all variants except of P21R and P21R-H159Y (Fig. 2c), most likely because P21R interferes strongly with BAFF binding and BAFFR oligomerization (Fig. 1c, d, [3]).

The SNPs encoding the different variants are mainly found in a heterozygous form resulting in the co-expression of variant BAFFRs and wildtype BAFFR. To test if the co-expression of variant and wildtype BAFFR would also change BAFFR oligomerization, we co-expressed GFP fusion proteins made with the different variants together with WT BAFFR-RFP and analyzed FRET before and after activation with BAFF for 1 h (Fig. 2d). Different from cells expressing only variant BAFFR, the percentages of resting or BAFF-activated FRET^+^ cells co-expressing WT BAFFR-RFP and G64V, A52T or P146S-GFP did not differ significantly from cells co-expressing WT BAFFR-GFP and BAFFR-RFP fusion proteins. The percentages of FRET^+^ cells were only significantly lower when P21R was co-expressed with WT BAFFR in both resting and BAFF-activated cells. Since the activation of BAFFR by BAFF requires clustering of BAFFR oligomers [27], these data support the genetic association studies which only showed in the case of P21R a positive correlation with CVID.

### BAFFR Variants Change BAFFR-Induced Signaling

#### Activation of NF-κB2

Binding of BAFF to BAFFR induces conformational changes promoting the recruitment of TRAF3 to the cytoplasmic part of BAFFR [28, 29]. This leads to the dissociation of NIK from TRAF3 and allows the phosphorylation of IKK1 and of NF-κB2 p100 by NIK. Phosphorylated NF-kB2 is then cleaved into active p52 which regulates the expression pro-survival genes [4].

We therefore analyzed the recruitment of TRAF3 in DG-75 BAFFR KO cells expressing WT or variant BAFFRs. Using FLAG-tagged BAFF to co-immunoprecipitate BAFFR, TRAF3 bound to BAFFR was detected by western blotting (Supplemental Figure S4). After 60 min of activation, significant differences to WT BAFFR were only detected in cells expressing P21R or H159Y. Since P21R binds BAFF less efficiently than WT BAFFR, less TRAF3 was found to co-immunoprecipitate with FLAG-BAFF. Differently, more TRAF3 was co-immunoprecipitated from lysates of H159Y-expressing cells, most likely because the H159Y mutation increases TRAF3 binding [22]. Although the P21R-H159Y double mutant binds BAFF almost as weakly as P21R, it was more efficient in recruiting TRAF3 (Fig. 3a), presumably because H159Y increased the affinity of the cytoplasmic tail of BAFFR to TRAF3 [22].

Next, we analyzed by western blotting if the differences between BAFFR and the variants in TRAF3 recruitment would be mirrored by changed processing of NF-κB2. Compared to DG-75 BAFFR KO cells, expression of BAFFR resulted in a higher “background” of processed NF-κB2 p52 in unstimulated cells (Fig. 3b,c). This background p52 signal was similar for all BAFFR variants except for H159Y, which seems to increase TRAF3 recruitment. Stimulation with BAFF activated processing of NF-κB2 in all cell lines except for P21R and P146S. Different from the enhanced recruitment of TRAF3, BAFF binding to H159Y did not induce significantly more NF-κB2 processing than WT BAFFR, indicating that binding of TRAF3 to BAFFR is an important but not the only rate-limiting step in activating NF-κB2. Like the increased recruitment of TRAF3, binding of BAFF to the P21R-H159Y double mutant also enhanced the generation of p52.

Thus, P21R impaired TRAF3 recruitment to BAFFR in response to BAFF. The P21R variant also interfered with BAFF-induced activation of NF-κB2, which was also slightly disturbed by P146S. Although H159Y increased spontaneous TRAF3 binding to BAFFR in activated cells, the variant did not induce in BAFF-activated cells processing of NF-κB2 above levels as those detected for WT BAFFR.

#### Interactions with CD79B

BAFF binding to BAFFR activates the PI3K signaling pathway, which regulates survival, differentiation, and cellular growth [8, 9]. BAFFR, however, lacks an immunoreceptor tyrosine-based activation motif (ITAM), which is found in the cytoplasmic tails of the B cell antigen receptor (BCR) components CD79A and CD79B [30], which has to be phosphorylated to initiate the activation of PI3K and AKT in response to antigen binding [31, 32]. To induce PI3K signaling, BAFFR therefore engages components of the BCR signalosome [33].

To test if the BAFFR variants would interfere with the interactions between BAFFR and BCR components, we expressed GFP fusion proteins of the different variants and WT BAFFR together with CD79B-RFP in DG-75 BAFFR KO cells. Ligand-independent and ligand-regulated interactions between BAFFR and CD79B were then analyzed by FRET applying a very similar approach as described above for the analysis of interactions between BAFFR molecules.

After lentiviral transduction of DG-75 BAFFR KO cells with BAFFR-GFP and CD79B-RFP expression vectors, CD79B-RFP and BAFFR-GFP single positive cells were used to adjust for the spillover signals generated by the emission of GFP and RFP into the channel detecting FRET-induced emission of RFP (Supplemental Figure S5a).

Co-expression of the WT BAFFR-GFP and CD79B-RFP revealed 26% FRET^+^ cells (Fig. 4b), indicating that BAFFR can spontaneously interact with CD79B. Stimulation with BAFF reduced the percentage to 18.2%, suggesting that BAFF binding to BAFFR induced a conformational change of the oligomeric complex leading to a more open or dissociated architecture. Four variants, P21R (18% FRET^+^ cells), G64V (14%), A52T (17%), and DUP92-95 (14%), interfered with spontaneous interactions between BAFFR-GFP and CD79B-RFP (Fig. 4b). Interestingly, three of them, P21R, A52T, and G64V, were also found to hinder ligand-independent BAFFR oligomerization (Fig. 2c). Although DUP92-95 (Supplemental Figure S5c, d) did not impair spontaneous BAFFR oligomerization (Fig. 2c), the variant inhibited spontaneous interactions of BAFFR and CD79B (Fig. 4b). Treatment with BAFF for 1 h reduced the percentage of FRET^+^ cells expressing P146S or H159Y similar to those expressing WT BAFFR indicating that the cytoplasmic tail of BAFFR was not involved in regulating interactions with CD79B. Differently, addition of BAFF did not change the percentages of FRET^+^ cells for P21R, A52T, G64V, and DUP92-95 (Fig. 4c) Thus, BAFFR oligomerization, which is disturbed by P21R, G64V, and A52T, and interactions between the TM regions of BAFFR and CD79B, which are affected by DUP92-95, seem to regulate spontaneous association of BAFFR and CD79B as well as BAFF-induced dissociation of the BAFFR-CD79B complex.

#### Activation of PI3K/AKT

BAFFR activates PI3K signaling by co-opting BCR components [33]. Since the variants P21R, A52T, G64V, and DUP92-95 changed the interactions between BAFFR and CD79B, we analyzed if they also would change the activation of PI3K. To this end, we measured BAFF-induced phosphorylation of AKT (S473) in time-course experiments (Fig. 4d, Supplemental Figure S6). WT BAFFR, P146S, and H159Y strongly induced AKT phosphorylation, whereas P21R, A52T, G64V, DUP92-95, and the P21R-H159Y double mutant responded much less to BAFF.

Thus, the pattern of interactions between BAFFR variants and CD79B—normal interactions of CD79B with P146S and H159Y, in contrast to weaker interactions with P21R, G64V, A52T, and DUP92-95—correlated with the intensity of BAFF-induced AKT phosphorylation: normal for P146S and H159Y and weak for P21R, G64V, A52T, and DUP92-95.

In primary B cells, activation of PI3K and AKT signaling leads to the phosphorylation of the small ribosomal protein S6, which can be detected by intracellular flow cytometry [34]. To determine if the variant-based changes in BAFF-induced AKT phosphorylation detected in the DG-75 model would correlate with changes in the phosphorylation of S6 in primary B cells, we activated PBMCs of CVID patients carrying P21R, G64V, DUP92-95, and H159Y with BAFF and analyzed pS6 (S240/244) by intracellular flow cytometry. Compared to matched controls carrying BAFFR WT alleles, the percentage of S6 (S240/244) phosphorylation was significantly weaker in B cells for carriers of the P21R and DUP92-95 variants but not in the case of the patient with the G64V and the H159Y variant (Supplemental Figure S7). Different from the activation with BAFF, stimulation with CD40L did not reveal differences in the percentage of pS6^+^ cells compared to healthy controls with WT BAFFR.

Thus, variants that disturb BAFF binding (P21R, A52T), ligand-independent (P21R, A52T, G64V) or BAFF-induced oligomerization of BAFFR (P21R), and interactions with CD79B (P21R, A52T, G64V, DUP92-95) also disturb BAFF-dependent PI3K signaling. In primary B cells of heterozygous carriers, the co-expression of WT BAFF proteins seemed to compensate the impairment resulting from G64V but not from P21R or DUP92-95 variants.

#### Activation of ERK1/2

BAFF-dependent B cell survival correlates with the ERK dependent turnover of the pro-apoptotic protein BIM [12, 13, 33]. Therefore, we investigated if the BAFFR variants would change BAFF-induced ERK phosphorylation (pT202/Y204) (Fig. 4e, Supplemental Figure S8). After 2 min of stimulation, lower ERK pT202/Y204 phosphorylation signals were detected for the variants P21R, G64V, and DUP92-95. Differently, A52T, P146S, H159Y, and P21R-H159Y activated ERK1/2 like WT BAFFR.

Thus, a common denominator of P21R, G64V, and DUP92-95 is the strong impairment of spontaneous interactions with CD79B. Since the inactivation of either CD79A or CD79B completely prevented BAFF-induced ERK1/2 phosphorylation (Fig. 4f), interactions between BAFFR and CD79B seem to be a central feature of BAFF-dependent ERK1/2 activation.

### BAFF-Induced Shedding of the BAFFR Ectodomain

BAFF binding to BAFFR induces proteolytic shedding of the BAFFR ectodomain by ADAM10 resulting in the downmodulation of BAFFR activity [35]. It can be detected by western blotting of total lysates by the decrease of total BAFFR levels and by the appearance of a signal of approximately 22 kDa in size corresponding the TM region and the cytoplasmic tail remaining inserted in the plasma membrane after processing of BAFFR.

We determined if the different BAFFR-variants would affect BAFFR processing by analyzing lysates of cells treated over night with BAFF by western blotting (Fig. 5a, Supplemental Figure S9). Only the P21R variants—P21R and P21R-H159Y—interfered with BAFF-induced processing of BAFF. This suggests that the BAFF-binding capacity has to decrease by 50% or more of WT BAFFR levels which was only the case for P21R (Fig. 1c). Therefore, BAFF binding to BAFFR in combination with BAFF-induced BAFFR-clustering seems to be two of the most important parameters regulating shedding of the BAFFR ectodomain.

**Fig. 6:**
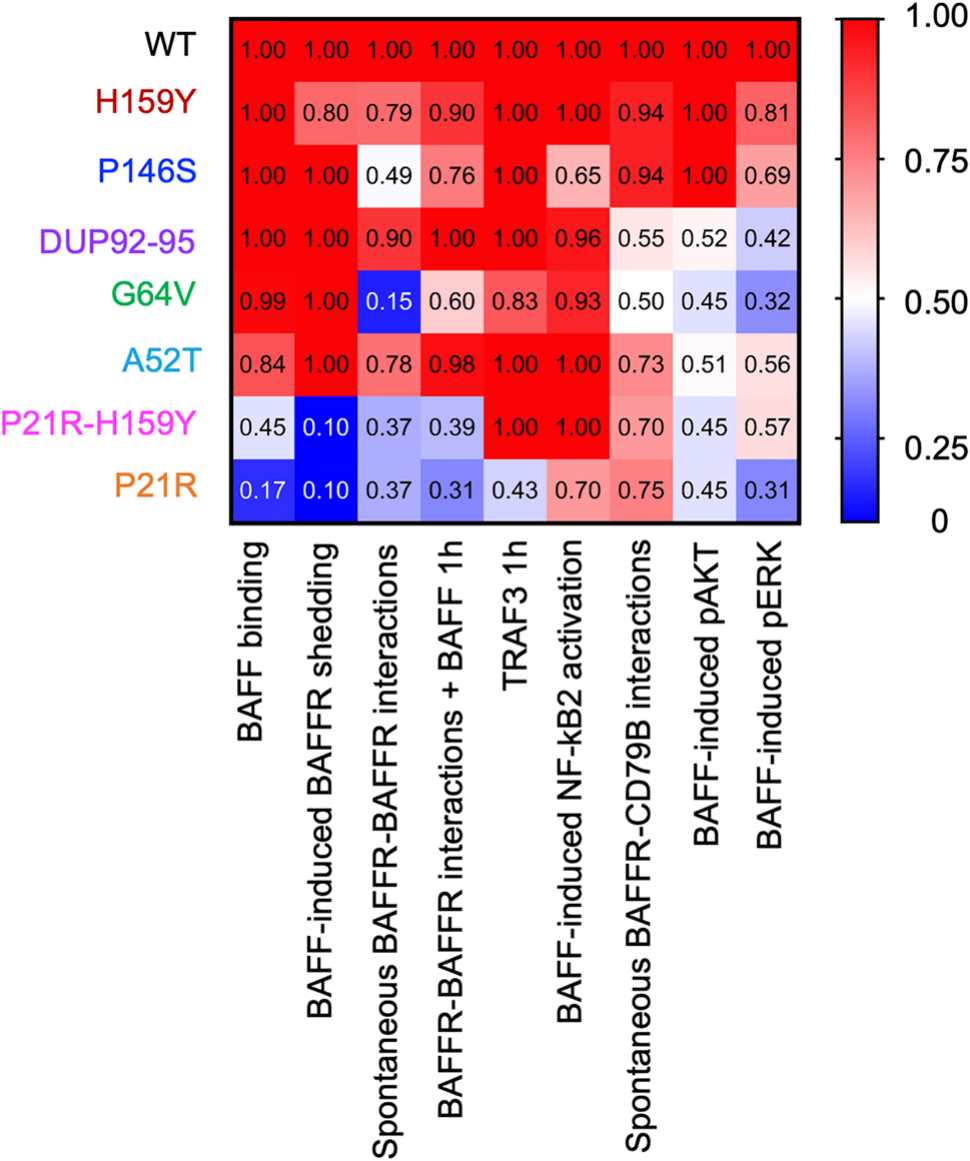
Comparison of WT and variant BAFFR. The heat map represents the grouped analysis of all experiments relative to WT BAFFR activity set a 1.0. It shows BAFF binding (from Fig. 1d), BAFF-induced shedding of BAFFR (from Fig. 5), the percentage of FRET^+^ cells before and 1 h after activation with BAFF (Fig. 2c, d), TRAF3 recruitment at *t* = 1 h (Fig. 3a), percentage of BAFF-induced NF-κB2 processing (Fig. 3b), the percentage of FRET.^+^ cells expressing BAFFR-GFP and CD79B-RFP in the absence of ligand (Fig. 4b), *n*-fold changes in pAKT at *t* = 5 min (Fig. 4d), and *n*-fold changes in pERK T202/Y204 at *t* = 2 min (Fig. 4e). WT BAFFR activity was set as 1.0 (red), 50% activity as 0.5 (white), and no BAFFR activity as 0 (blue)

In summary, our data show that the different variants affect BAFFR function by changing ligand-independent as well as with ligand-dependent oligomerization of BAFFR, BAFF-induced activation of BAFFR, the association of BAFFR with CD79B, and constitutive receptor activity (Fig. 6).

## Discussion

The *BAFFR* variants P21R, G64V, and H159Y have been described in patients with common variable immunodeficiency [19]. Since BAFFR activates pro-survival functions, it has been proposed that these variants contribute to the development of CVID and antibody deficiencies [36], lymphoma [22, 37, 38], or autoimmune diseases [23, 39]. In addition to these variants, we also describe here two variants—A52T and DUP92-95—that were newly found in CVID patients.

Except for P21R and H159Y, it has remained unclear to which extent such BAFFR variants change BAFFR function and if they correlate with or contribute to CVID. In our approach, we analyzed their allelic frequencies in CVID patients and studied their impacts on BAFFR function in a cellular model, which is based on the expression of the variants in the Burkitt’s lymphoma cell line DG-75.

By a functional step-by-step analysis, we first tested if the variants differ in binding to BAFF. Since BAFFR assembles spontaneously into homo-oligomers [3], we then analyzed if the variants interfere with ligand-independent oligomerization. Binding of BAFF to BAFFR crosslinks BAFFR oligomers and initiates BAFFR signaling [3, 27]. We therefore examined changes in BAFF-dependent BAFFR clustering as well as the activation of NF-κB, PI3K/AKT, and ERK1/2 signaling pathways. Since BAFF-induced activation of PI3K/AKT and of ERK1/2 involves components of the B cell antigen receptor [33], we analyzed if the variants would affect interactions between BAFFR and CD79B. BAFF binding to BAFFR does not only induce BAFFR signaling, but it also activates the shedding of the BAFFR ectodomain by ADAM10/17 proteases [35]. We therefore tested if the variants would impair BAFFR processing.

Of all variants, only the P21R missense mutation impaired all functions—binding of BAFF, ligand-independent and ligand-induced oligomerization, ectodomain shedding, recruitment of TRAF3 and activation of NF-κB2, interactions with CD79B and activation of AKT and ERK1/2 signaling. The P21 residue lies within the pre-ligand assembly domain [3], which forms together with the ligand binding domain the single CRD of BAFFR. According to its 3D structure [40], the PLAD points away from the ligand binding loop, and therefore, P21R is unlikely to interfere directly with BAFF binding. But because ligand-independent oligomerization greatly enhances ligand binding [3], this variant also affects BAFF-induced downstream activation of NF-κB2, ERK1/2, and PI3K/AKT. This impairment of BAFFR functions is reflected by the significant association of the P21R encoding SNP rs77874543 with CVID (*P* = 0.0145 – 0.0458). Since rs77874543 has an allele frequency of 8.6% in the general population, it remains an open question, why it is found with this high frequency in spite it affects BAFFR responses even in the heterozygous situation when co-expressed with WT BAFF. One explanation would be that P21R affects B cell responses not enough to cause a selective disadvantage, but at least for T-independent B cell activation this seems not to be the case [3]. Alternatively, P21R could dampen overshooting or autoimmune B cell responses, which—as shown in the course of SARS-CoV-2 infections—can enhance infection [41] and account for > 20% of deaths in certain populations ([42, 43].

The P21R encoding rs77874543 co-segregates 10–15 × more frequently than expected with rs61756766 encoding H159Y. H159Y increases TRAF recruitment as well as activation of NF-κB2, which correlates positively with the development of lymphoma and autoimmunity [20, 22, 23]. Our experiments show that H159Y functions like WT BAFFR except for the enhanced TRAF3 recruitment and NF-κB2 activation. The combination of P21R and H159Y displayed features of both variants: impaired BAFF binding, ligand-independent as well as BAFF-induced oligomerization, BAFFR processing, interaction with CD79B and AKT phosphorylation like P21R, but normal TRAF3 recruitment, NF-κB2 activation, and ERK1/2 phosphorylation like H159Y. Different from P21R, co-expression of WT BAFFR increased BAFF-induced clustering of P21R-H159Y suggesting that in a heterozygous situation, B cells of P21R-H159Y carriers would respond normally to BAFF-induced activation. This assumption correlates with the frequency for P21R-H159Y in CVID patients which does not differ significantly from the control population (*P* = 0.8233). Thus, the linkage disequilibrium observed for P21R-H1159Y could reflect a selective advantage of rs77874543 (P21R) carriers who have acquired the H159Y mutation because the mutation would balance some of the defects of P21R and allow close to normal B cell responses.

G64V and A52T reside in the loop from Leu37-Leu71 connecting the CRD to the TM region, which is affected by DUP92-95. A52T slightly reduced BAFF binding, both G64V and A52T disturbed ligand-independent but not ligand-dependent BAFFR oligomerization, and all three variants interfered with ligand-independent as well as ligand-dependent interactions between BAFFR and CD79B. Weaker interactions of A52T, G64V, and DUP92-95 with CD79B correlate with impaired phosphorylation of AKT and, in the case of G64V and DUP92-95, also with reduced phosphorylation of ERK1/2 in BAFF-activated cells. Thus, the loop region Leu37-Leu71 as well as the TM region could be involved in contacts between BAFFR and CD79B. CD79B forms a heterodimer with CD79A and both interact closely with the heavy chains of surface immunoglobulins through their extracellular parts and transmembrane domains [44–48]. The TM region of BAFFR contains several leucines which could form two leucine zippers located on opposite sites of the TM α-helix. These zippers could provide the interfaces for the contacts in between TM regions of BAFFR chains as well as with the TM region of CD79B. The DUP92-95 reduces the leucin zipper L(X)_6_L repeats within the TM region from four to three. Since the duplication does not change ligand-independent BAFFR oligomerization, this part of the BAFFR TM region could be the side of the TM region involved in ligand-independent contacts with CD79B.

The G64V encoding rs547352394 allele was so far found in CVID patients in a heterozygous form. Since the co-expression of G64V with WT BAFFR restored ligand-independent oligomerization, the cellular model predicts that in a heterozygous situation B cells of G64V carriers respond normally to BAFF. This was supported by normal phosphorylation of S6 in BAFF-activated B cells from a heterozygous G64V carrier, reflecting the finding that rs547352394 does not associate with CVID.

A52T was found so far not in the general population and only once in a heterozygous form in a CVID patient who was treated with rituximab because of severe autoimmune cytopenia. A52T reduced BAFF binding to 83% of WT BAFFR levels and impaired ligand-independent BAFFR oligomerization, interactions with CD79B, and phosphorylation of AKT and ERK1/2. Therefore, it seems unlikely that this variant was responsible for the development of autoimmunity in this patient because autoimmunity would be expected to correlate with increased and not with reduced B cell functions.

DUP92-95 was found only in one CVID patient and was inherited from the unaffected mother indicating its incomplete penetrance. However, B cells of both individuals did not respond to BAFF by phosphorylating S6, and in the DG-75 model system, the DUP92-95 variant also impaired AKT and ERK1/2 phosphorylation. Since DUP92-95 disturbed ligand-independent and ligand-dependent interactions between BAFFR and CD79B, the TM region seems to form an interface between BAFFR and CD79B that allows BAFFR to fully activate PI3K signaling via the BCR component. As it was found only once in a CVID patient and because the corresponding SNP rs776259962 is very rare (1/234974), we cannot conclude if this variant associates with CVID.

P146S has not yet been discovered in CVID patients. The variant affects the first proline of a highly conserved proline-rich motif (WPPPG) within the intracellular region of BAFFR. After P21R (8.67% allele frequency), G64V (0.9%), H159Y (0.75%), and R106Q (0.15%), P146S is the fourth most common BAFFR variant. It affected activation of NF-κB2 and ligand-independent BAFFR oligomerization as seen by the reduced percentage of FRET^+^ cells. Lower percentages of FRET^+^ cells could be result from structural changes of the cytoplasmic tail which would increase the distance between the GFP and RFP parts of the fusion proteins, while reduced activation NF-κB2 could reflect TRAF3 recruitment that remained undetected by the BAFFR-TRAF3 co-immunoprecipitation experiments.

## Conclusion

Our data provide evidence that the BAFFR variants P21R, A52T, G64V, DUP92-95, P146S, and H159Y impair BAFFR function in relation to their position in the polypeptide chain. Except for P21R, these changes can be balanced in heterozygous individuals by the co-expression of WT BAFFR. Thus, P21R seems so far to be the only reported BAFFR variant that disturbs BAFFR functions strong enough to correlate positively with CVID.

## Supplementary Information

Below is the link to the electronic supplementary material.

Supplementary file1 (DOCX 10796 KB)

## Data Availability

Data and material will be made available when requested in agreement with a respective MTA.
