# Supplementary material for: CVID-Associated B Cell Activating Factor Receptor Variants Change Receptor Oligomerization, Ligand Binding, and Signaling Responses
Source: J Clin Immunol. 2022 Oct 29;43(2):391–405. doi: 10.1007/s10875-022-01378-3 (PMC9616699; doi:10.1007/s10875-022-01378-3)
Supplement: Supplementary file 1 — Supplementary file1 (DOCX 10796 KB) [file 10875_2022_1378_MOESM1_ESM.docx]

Supplemental information

CVID-associated B cell activating factor receptor variants change receptor oligomerization, ligand binding and signaling responses

Violeta Block^1,2^, Eirini Sevdali^1,2^, Mike Recher^3^, Hassan Abolhassani^4,5^, Lennart Hammarstrom^4^, Cristian R. Smulski^1,6^, Manuela Baronio^6^, Alessandro Plebani^6^, Michele Proietti^7,2^, Matthaios Speletas^8^, Klaus Warnatz^1,2^, Reinhard E. Voll^1,2^, Vassilios Lougaris^6^, Pascal Schneider^9^, Hermann Eibel.^1^

^1^ Dept., of Rheumatology and Clinical Immunology, Medical Center and Faculty of Medicine, University of Freiburg, Germany

^2^ Center for Chronic Immunodeficiency, Medial Center and Faculty of Medicine, University of Freiburg

^3^ Immunodeficiency Clinic and Laboratory, Medical Outpatient Unit and Department Biomedicine, University Hospital and University of Basel, Switzerland

^4^ Department of Biosciences and Nutrition, Karolinska institutet, Huddinge, Sweden

^5^ Research Center for Immunodeficiencies, Pediatrics Center of Excellence, Children's Medical Center, Tehran University of Medical Sciences, Tehran, Iran

^6^ Medical Physics Department, Centro Atómico Bariloche, Comisión Nacional de Energía Atómica (CNEA), Consejo Nacional de Investigaciones Científicas y Técnicas (CONICET), San Carlos de Bariloche, Río Negro, Argentina

^6^ Dept. of Clinical and Exp Sciences, University of Brescia, Italy

^7^ Institute for Immunodeficiency, Medical Center and Faculty of Medicine, University of Freiburg, Germany

^8^ Department of Immunology & Histocompatibility, Faculty of Medicine, University of Thessaly, Larissa, Greece

^9^ Department of Biochemistry, University of Lausanne, Epalinges, Switzerland^.^

*Correspondence: [hermann.eibel@uniklinik-freiburg.de](mailto:hermann.eibel@uniklinik-freiburg.de)

Hermann Eibel ORCID: 0000-0003-2901-2809

# Supplemental methods

## Subjects and cell lines

Human embryonic kidney HEK293T cells and the human Burkitt lymphoma B cell line DG-75 were cultivated under sterile conditions at 37°C and 5% CO2 in Iscove’s medium (Gibco) containing L-glutamine and HEPES, supplemented with 10% heat-inactivated fetal calf serum (FCS, Sigma Aldrich) and 100U/ml penicillin/streptomycin (P/S). The DG-75 cell lines carrying the different BAFFR variants were generated by lentiviral transduction of DG75 BAFFR KO cells. The work with human samples was performed in accordance with the ethical standards of the 1964 Helsinki declaration and the approvals 169/13 and 428/17 including all amendments by the Ethics Commission of the Albert-Ludwigs-University of Freiburg to HE. All donors agreed by informed consent.

## Genetic analysis of BAFFR variants

Genomic DNA was extracted from whole blood by standard methods and whole exome sequencing (WES) was performed as reported before (1-4). For analysis of WES, we followed the protocol described previously for prioritizing candidate variants, predicting their effect on protein, homozygosity mapping, large deletion and copy number variation detection (5, 6). The pathogenicity of all disease attributable gene variants was re-evaluated using the updated guideline for interpretation of molecular sequencing by the American College of Medical Genetics and Genomics (ACMG), considering the allele frequency in the population database, computational data, immunological/functional data, familial segregation and parental data and clinical phenotyping (7). For the identified BAFFR variant, 20 ng of genomic DNA was used in Sanger sequencing and segregation analysis of patients and their family members employing exon-specific primers (5’-CTTCAGCCAAACTTGCAGCTC and 5’- CCTCTTTGCAATGGTGTCCAC). The heterozyous BAFFR c.G154A;p.A52T mutation was detected in PBMC derived genomic DNA by whole exome sequencing performed as recently described (8). The mutation was confirmed by Sanger PCR using the following BAFFR exon 2 primers 5’TGTCCCCTCCCGAAGCAG and 5’-TTCCCCTACACAGGAACTC. 5% DMSO was added to the PCR reaction, annealing temperature was 60°C.

The P21R and H159Y variants were identified by restriction site polymorphisms as described (9, 10).

## CRISPR/Cas9 site-directed mutagenesis

The cRNA 5’-AACCGGGUAAGGGGGACCCACGG to target *TNFRSF13C* and the cRNAs 5-’UCCUCCAUGGCAACUACACGUGG to inactivate *CD79A* and 5’-A: AACACCUCGGAGGUCUACCAGGG (PAM sequences are underlined) to mutate *CD7B* were purchased from Integrated DNA Technologies (IDT).

The guide RNA (gRNA) was formed by combining equimolar amounts of 200µM crRNA and tracer RNA (tracrRNA, IDT) in IDTE duplex buffer to a final concentration of 44µM, followed by a 5min incubation at 95°C. For the formation of the ribonucleoprotein (RNP) complex the gRNA was mixed in a 1:1 ratio with 36µM Cas9 enzyme and incubated for 20min at RT. Prior to electroporation, 5 x 10^5^ DG75 cells were washed twice with PBS (256xg; 5min; RT) and resuspended in 9µl Neon electroporation R buffer. The cell suspension was added to the RNP complex and mixed with 10.8µM electroporation enhancer (IDT). For each electroporation reaction, cells were loaded together into a 10µl neon tip and inserted in the neon tube containing electrolytic buffer. The Neon transfection system (Invitrogen) was at 1350V, 30ms, 1 pulse. Afterward, the cells were recovered in Icove’s medium supplemented with 20% FCS without antibiotics for 24-48h

## Construction of BAFFR expression vectors

Before the subcloning of the BAFFR variants into the lentiviral expression vectors pNL-CEF-GS-GFP and pNL-CEF-GS-RFP, the vectors were cleaved with EcoRI and SalI. The different PCR fragments encoding BAFFR constructs were amplified using the Q5 site-directed mutagenesis kit according to the manufacturer’s protocol. For the pNL-CEF expressing BAFFR variants (A52T, G64V and Dup92-95), the forward primer 5’-CCCAAGCTGGCTAGCGTTTGAATTCCACCATGAGGCGAGGGC and reverse primer 5’-GAGCTGTGGCATCAGAGATTCC were used. In the case of the BAFFR variants (P21R, P146S and H159Y) encoded by retroviral MIGR-based expression vectors (9), the BAFFR-MIGR1 primers 5’-CAAGCTGGCTAGCGTTTGACCATGAGGCGAGGGC and 5’-GATCCGCTGCCTGGTCCGCCTTGTTGCTCAGGGCC were used.

Vectors and insert DNA was separated by agarose gel electrophoresis. Fragments were isolated with the NucleoSpin Gel and PCR clean-up kit (Thermofisher). DNA fragments were ligated with the NEBuilder HiFi DNA Assembly kit (New England Biolabs) for 15min at 50°C, according to the manufacturer’s instructions. STBL2 competent cells were inoculated in 2 ml LB containing 75µg/ml ampicillin overnight at 37°C. DNA was purified using the NucleoSpin Plasmid EasyPure kit (Macherey-Nagel) according to manufacturer protocol. The amino acid exchanges in BAFFR were confirmed by DNA sequencing with Mix2 sequencing kit (Eurofins Genomics) using 5’-CACACTGAGTGGGTGGAGAC in combination wiith 5’-GGATGCCCTGGGTGTGGTTGAT or 5’-CGGCGGCGGTCACGAACTCC as primers.

## Lentiviral gene transfer

3 x 10^5^ 293T cells were seeded one day before transfection into a 6-well plate in 2ml complete culture medium to obtain 60-80% confluency. Transfections were carried out using JetPEI reagent (Polyplus-transfection) according to the manufacturer’s instructions, with 0.9µg packaging plasmid (pCD/NL-BH*), 0.9µg envelope plasmid (pLTR-VSV-G) and 1.5µg target DNA encoding for the different BAFFR constructs as described before (9, 11). After two days of cultivation, the supernatant containing virus particles was collected, centrifuged (256xg; 7min, RT) filtered through 0.45µm syringe filter and pelleted (3600 x g, 16h, 4°C). The concentrated virus was resuspended in cell cultire medium at 1/20 of the starting volume was added to 3 x 10^4^ DG75 BAFFR KO cells and incubated for 1h, followed spin infection (1111 x g; 120min, 30°C).

## PBMC isolation

Peripheral blood mononuclear cells (PBMCs) were isolated from whole blood by Ficoll density gradient centrifugation (9, 11). Blood samples were diluted 1:2 in sterile PBS supplemented with 2mM EDTA (PBS-EDTA) and layered over 20ml Ficoll (PanBiotech) in 50ml Falcon tubes. After centrifugation (1000 x g; 15min; RT), PBMCs were isolated from the interphase and washed twice (585 x g; 7min; RT) with PBS-EDTA. Cells were then counted and kept in culture for further analysis or were frozen at -80°C.

## Flow cytometry

Flow cytometry was performed as described (9, 11, 12). 2 x 10^4^ – 2 x 10 cells / sample in 50µl PBS supplemented with 5% FCS and 2mM EDTA ("FACS buffer") were incubated with fluorochrome-conjugated antibodies diluted to the appropriate concentration in the dark for 15 min at 4°C. The following antibodies were used: anti-CD19 APC-Cy7 (Biolegend), anti-CD27 PE-Cy7 (Biolegend), anti-IgD FITC (SouthernBiotech), anti-BAFFR PE or APC (Biolegend). anti-pS6 PE (Cellsignal), anti-CD79A PE (Biolegend) and anti-CD79B APC (Biolegend). FACS buffer was added to 200 µl and cells were washed (256xg; 5min, 4°C). Cell pellets were resuspened in FACS buffer containing 100ng/ml 4′,6-Diamidino-2-phenylindole (DAPI, eBioscience) to discriminate living from dead cells. After a second washing step, cells were resuspended in 200 µl FACS buffer and analyzed using a FACS Canto II Flow cytometer and FACS Diva (BD Biosciences). The FCS files containing the data were analyzed with FlowJo (BD Biosciences). For intracellular flow cytometry, cells were first fixed with 4% paraformaldehyde for 20 min at room temperature in the dark. After pelleting the cells by centrifugation (256xg; 10 min, RT) and removal of the supernatant, cells were permeabilized by for 20 min in the dark in 0.1% saponin-PBS at RT.

## Flow cytometry-based FRET

FRET was performed as described (9, 12, 13). DG75 BAFFR KO cells were co-transduced with lentiviral vectors carrying CD79B, BAFFR WT or the different BAFFR variants fused at the C-terminal domain to GFP or RFP. After 1h of stimulation with 100 ng/ml BAFF, 2 x 10^5^ cells were washed (256xg; 5min; RT) and resuspended in 100µl Fluorobrite DMEM medium (Thermo Fischer Scientific) containing 5% FCS and analyzed in parallel to untreated cells on a LSRII Fortessa flow cytometer (BD Biosciences). The donor fluorophore (GFP) signal was measured using the 488nm blue laser with a 505 long pass (LP) and 530/30 bandpass (BP) filters. RFP was generated by the 561nm yellow/green laser and detected using a 570 LP and 586/15 BP filters. The FRET signal (GFP-RFP) was the result of RFP excited by GFP and collected by the 595 LP and the 625/15nm BP filters. Single GFP- or RFP-positive cells were used to determine the spillover of GFP or RFP into the FRET channel as described (12). The data were analyzed with FlowJo (BD Bioscience).

## Ligand-binding assay

Active Flag-ACRP-ligands have been described (13). The plasmid for Flag-ACRP-hBAFF, thereafter called Flag-BAFF, encoded HA signal-Flag-GPGQVQLH-mACRP30 (18-111)-L-hBAFF (136-285), where HA signal = MAIIYLILLFTAVRG, and FLAG = DYKDDDDK. Flag-BAFF in conditioned, serum-free OptiMEM supernatants of transiently transfected HEK-293T cells was used at a starting concentration sufficient to saturate the staining of DG-75 cells by FACS. 2 x 10^5^ cells were incubated with 50µl FLAG-BAFF in 1:1 serial dilutions ranging from 18.75.ng/ml – 1200 ng/ml for 30 min at 4°C. Cells were washed (256 x g; 5min; 4°C) with FACS buffer and stained for 30 min on ice with 50µl anti-FLAG antibody diluted in FACS buffer. After two washing steps (256 x g; 5min; 4°C) samples were acquired on FACS Canto II Flow cytometer (BD Biosciences) and dose dependent ligand binding was analyzed with Graphpad Prism 9 assuming one site-specific binding.

## Western blot analysis

Whole-cell lysates of 10^6^ human B cells or of 2.5 x 10^5^ DG-75 cells / sample were prepared as described (11). Proteins were separated by sodium dodecyl sulfate- polyacrylamide gel electrophoresis (SDS-PAGE) using 10% polyacrylamide at 80V for 3h. The transfer of proteins into a nitrocellulose membrane (0.2µM, Bio-Rad) was performed at 100 V for 1h. The membrane was blocked for 1h at RT in PBS containing 0.1% Tween (PBS-Tween) supplemented with 5% non-fat dry milk. The following primary antibodies were used to incubate the membranes overnight at 4°C: anti-BAFFR CT (Enzo), anti-TRAF3 (Cell Signaling), anti-NF-κB2 (Millipore), anti-phospho-AKT (Ser 473, clone D9E, Cell Signaling), anti-phospho-p44/42 MAPK (Thr202/Tyr204, Cell Signaling) and anti-beta actin (clone AC15, Sigma Aldrich). The next day, membranes were washed with PBS-Tween and incubated for 1h while shaking with horseradish peroxidase (HRPO)-coupled donkey anti-mouse IgG and donkey anti-rabbit IgG secondary antibodies (Jackson Immuno Research). After 5 washing steps with PBS-Tween, the chemiluminescent signal was detected by applying SuperSignal West Pico Chemiluminescent Substrate (Thermo Fischer Scientific) or WesternBright Quantum HRP substrate (Advansta) using the Fusion Fx (Vilber Lourmat) and the Fusion Capt Advance Software (Vilber Lourmat). The differences in protein expression were quantified by densitometric analysis with ImageJ (NIH).

## Co-immunoprecipitation

Co-immunoprecipitation was carried out as described before (11).

2 x 10^7^ DG75 cells were stimulated with FLAG-BAFF in a time course (0, 15, 60min). Cells were then washed once with ice-cold PBS and lysed for 15min in 500µl lysis buffer (0.2% NP-40, 20 mM Tris-HCl, 150mM NaCl, 10% glycerol, complete protease inhibitor cocktail). Cell lysates were centrifuged at 13000 rpm for 10 min at 4°C. 450µl of the supernatants were transferred into Eppendorf tubes containing 50µl anti-FLAG magnetic beads (Pierce™ DYKDDDDK Magnetic Agarose, Thermo Fischer) that were previously washed and equilibrated with lysis buffer. After incubation at 4° for 4 h, the samples were placed in magnets to separate unbound proteins and washed twice with 200µl lysis buffer. Next, the immunoprecipitated protein complexes were eluted with 60µl citrate buffer (pH 2.4) and neutralized with 15µl 1.5M Tris buffer (pH 9). For western blot detection, Laemmli sample buffer was added to the samples before they were vortex and heated at 95°C for 5 min.

## Fluorescence microscopy

3 x 10^4^ cells were resuspended in 100µl Fluorobrite DMEM medium (Thermo Fischer Scientific) containing 5% FCS and transfer to a collagen-coated 4 well microscopy dish (Greiner). Images were taken in a 63X magnification in GFP, RFP and DAPI channels with a Axio Observer (Zeiss) inverted micrcoscope equipped with an incubation chamber at 6.5% CO_2_ athmosphere. The data were analyzed with Zen software (Zeiss).

## Structural models

All structural models of BAFFR were made with UCSF Chimera, UCSF Modeller (14-17) and Swiss model (18). They are based on the 3D model of BAFFR (https://www.alphafold.ebi.ac.uk/entry/Q96RJ3) generated by Alphafold (19).

## Statistical analysis

Statistical analysis was performed with Prism 9 (GraphPad). Multiple comparisons of data presented in colums were made with Brown-Forsythe and Welchs ANOVA assuming unequal SD applying the Dunnet T3 corection for n<50/group with P≥0.1234 (ns); P≤0.0032 (*); P≤0.0021 (**); P≤0.0002 (***); P<0.00001 (****).

# Supplemental Table S1 Alignement of BAFFR proteins of 21 species.

|  |  |  |  |  |  | 1 |  |  |  |  |  |  |  | 1 | 0 |  |  |  |  |  |  |  |  | 2 | 0 |  |  |  |  |  |  |  |  | 3 | 0 |  |  |  |  |  |  |  |  | 4 | 0 |  |  |  | 4 | 4 |
| --- | --- | --- | --- | --- | --- | --- | --- | --- | --- | --- | --- | --- | --- | --- | --- | --- | --- | --- | --- | --- | --- | --- | --- | --- | --- | --- | --- | --- | --- | --- | --- | --- | --- | --- | --- | --- | --- | --- | --- | --- | --- | --- | --- | --- | --- | --- | --- | --- | --- | --- |
| Q96RJ3 HUMAN |  |  |  |  |  | M | R | R | G | P | R | S | L | R | G | R | D | A | P | A | P | T | P | **C** | V | **P** | A | E | **C** | F | D | L | L | V | R | H | **C** | V | A | **C** | G | L | L | R | T | P | R | . | P | K |
| H2QLT1 CHIMPANZEE |  |  |  |  |  | M | R | R | G | P | R | S | L | R | G | R | D | A | P | A | P | T | P | **C** | V | **P** | A | E | **C** | F | D | L | L | V | R | H | **C** | V | A | **C** | G | L | L | R | T | P | R | . | P | K |
| G3R8S3 GORILLA |  |  |  |  |  | M | R | R | G | P | R | S | L | R | G | R | D | A | P | A | P | T | P | **C** | V | **P** | A | E | **C** | F | D | L | L | V | R | H | **C** | V | A | **C** | G | L | L | R | T | P | R | . | P | K |
| A0A5F7ZFG6 MACAQUE |  |  |  |  |  | M | K | R | G | P | R | S | L | R | G | R | D | A | P | A | P | T | P | **C** | V | **P** | A | E | **C** | F | D | L | L | V | R | H | **C** | V | A | **C** | G | L | L | R | T | P | R | . | P | K |
| H9KVT2 MARMOSET |  |  |  |  |  | M | R | R | G | P | R | S | L | R | G | R | D | A | P | V | P | T | P | **C** | V | **P** | T | E | **C** | Y | D | L | L | V | R | K | **C** | V | D | **C** | R | L | L | R | R | S | P | . | P | K |
| H0XRB7 OTELMUR GARNETTI |  |  |  |  |  | M | Q | R | G | A | R | R | P | R | G | R | D | G | P | V | P | T | S | **C** | Y | **P** | A | Q | **C** | F | D | P | L | L | R | L | **C** | V | D | **C** | E | L | F | R | T | P | E | S | P | A |
| G3U9C5 AFRICAN ELEPHANT |  |  |  |  |  | M | G | L | G | S | R | S | M | G | G | R | D | G | S | E | P | T | L | **C** | G | Q | A | E | **C** | F | D | L | L | L | R | V | **C** | V | A | **C** | R | L | F | R | T | P | E | . | P | S |
| H0W812 GUINEA PIG |  |  |  |  |  | M | G | P | G | S | R | S | L | K | G | M | S | G | P | A | P | T | R | **C** | V | Q | T | E | **C** | F | D | H | L | V | R | H | **C** | V | S | **C** | R | L | L | R | T | P | D | . | . | . |
| A0A6J2A960 CHEETAH |  |  | M | G | S | Q | R | R | G | A | R | G | L | R | G | R | E | G | A | A | P | T | K | **C** | V | V | D | K | **C** | F | D | P | L | V | R | K | **C** | V | D | **C** | R | L | L | R | T | P | E | . | P | G |
| A0A6P6I3X8 PUMA |  |  | M | G | S | Q | R | R | G | A | R | S | L | R | G | R | E | G | S | A | P | T | K | **C** | V | V | D | K | **C** | F | D | P | L | V | R | K | **C** | V | D | **C** | R | L | L | R | T | P | E | . | P | G |
| M3YY53 EUROPEAN POLECAT |  |  | M | G | S | K | R | R | G | A | R | N | R | R | G | R | D | S | P | A | P | T | Q | **C** | V | Q | A | Q | **C** | F | D | P | L | V | R | N | **C** | V | A | **C** | N | L | F | R | T | P | E | . | P | R |
| A0A3Q7UGN9 GRIZZLY BEAR |  |  | M | G | S | K | R | R | G | A | R | S | L | R | G | R | D | G | P | A | P | T | Q | **C** | V | Q | A | E | **C** | F | D | P | L | V | R | N | **C** | V | A | **C** | K | L | F | R | T | S | E | . | P | R |
| A0A2U3ZCW5 WALRUS |  |  | M | G | S | K | R | R | G | A | R | S | R | R | G | R | D | G | P | A | P | T | Q | **C** | V | Q | A | E | **C** | F | D | P | L | V | R | N | **C** | V | A | **C** | K | L | F | R | T | P | E | . | P | R |
| A0A1S2ZRM0 EUROP. HEDGEHOG |  |  |  |  |  | M | R | R | . | . | R | G | R | R | S | R | D | G | Q | A | P | S | P | **C** | L | E | A | L | **C** | F | D | L | L | V | R | D | **C** | V | A | **C** | S | F | L | H | T | S | E | . | P | R |
| F1MJD0 CATTLE |  |  |  |  |  | M | Q | R | G | R | R | S | L | R | G | K | D | R | P | A | P | T | Q | **C** | L | Q | T | Q | **C** | F | D | P | L | V | R | N | **C** | V | A | **C** | S | L | L | R | T | T | G | . | P | R |
| A0A452EY37 GOAT |  |  |  |  |  | M | P | R | G | R | R | S | L | R | G | K | D | R | P | A | P | T | Q | **C** | L | Q | T | Q | **C** | F | D | P | L | V | R | N | **C** | V | A | **C** | S | L | L | R | T | T | E | . | P | R |
| A0A2Y9T0B2 SPERM WHALE | M | G | F | G | R | M | R | R | G | P | R | G | L | R | G | R | D | R | P | A | A | P | Q | **C** | L | Q | T | Q | **C** | F | D | P | L | V | R | K | **C** | V | A | **C** | S | L | L | R | T | T | E | . | P | R |
| A0A6P3QWA8 FLYING FOX |  |  |  |  |  | M | R | R | G | A | R | S | P | R | G | R | D | A | P | A | P | T | Q | **C** | V | Q | A | Q | **C** | F | D | P | L | V | R | N | **C** | V | A | **C** | K | L | F | R | T | P | E | . | P | G |
| A0A287AJ47 PIG |  |  |  |  |  | M | R | R | R | A | R | S | L | R | G | R | D | G | P | V | P | T | Q | **C** | L | Q | T | Q | **C** | F | D | L | L | V | R | N | **C** | V | A | **C** | S | L | L | R | T | P | D | . | P | G |
| Q9D8D0 MOUSE |  |  | M | G | A | R | R | L | R | V | R | S | Q | R | S | R | D | S | S | V | P | T | Q | **C** | N | Q | T | E | **C** | F | D | P | L | V | R | N | **C** | V | S | **C** | E | L | F | H | T | P | D | . | . | . |
| Q3BK46 CHICK |  |  |  | M | Q | E | R | S | A | M | A | S | P | G | K | A | D | G | G | A | S | . | . | **C** | L | S | S | Q | **C** | F | D | P | L | T | R | S | **C** | V | M | **C** | S | E | L | F | G | D | N | T | T | D |

|  | 4 | 5 |  |  |  |  |  | 5 | 0 |  |  |  |  |  |  |  |  | 6 | 0 |  |  |  |  |  |  |  |  |  |  |  |  |  | 7 | 0 |  |  |  |  |  |  |  |  |  | 8 | 0 |  |  |  | 8 | 5 |
| --- | --- | --- | --- | --- | --- | --- | --- | --- | --- | --- | --- | --- | --- | --- | --- | --- | --- | --- | --- | --- | --- | --- | --- | --- | --- | --- | --- | --- | --- | --- | --- | --- | --- | --- | --- | --- | --- | --- | --- | --- | --- | --- | --- | --- | --- | --- | --- | --- | --- | --- |
| Q96RJ3 HUMAN | P | . | . | A | G | A | . | S | S | P | A | P | R | T | A | L | Q | P | Q | E | S | V | **G** | A | G | A | G | . | . | . | . | . | E | A | A | L | . | P | L | P | G | L | L | *F* | *G* | *A* | *P* | *A* | *L* | *L* |
| H2QLT1 CHIMPANZEE | P | . | . | A | G | A | . | S | S | P | A | P | R | T | A | L | Q | P | Q | E | S | V | **G** | A | G | A | G | . | . | . | . | . | E | A | A | L | . | P | L | P | G | L | L | *F* | *G* | *A* | *P* | *A* | *L* | *L* |
| G3R8S3 GORILLA | P | . | . | G | K | G | . | D | . | . | . | P | R | G | . | . | . | P | Q | E | S | V | **G** | A | G | A | G | . | . | . | . | . | E | A | A | L | . | P | L | P | G | L | L | *F* | *G* | *A* | *P* | *A* | *L* | *L* |
| A0A5F7ZFG6 MACAQUE | P | . | . | A | P | A | . | S | S | P | **A** | P | R | T | A | L | Q | P | Q | E | S | V | **G** | A | G | A | G | . | . | . | . | . | E | A | A | L | . | S | L | P | G | L | L | *F* | *G* | *A* | *P* | *A* | *L* | *L* |
| H9KVT2 MARMOSET | T | . | . | A | G | A | . | S | S | P | **A** | P | G | T | A | L | Q | P | Q | E | S | V | **G** | P | G | A | G | . | . | . | . | . | E | V | S | L | . | P | V | P | R | L | L | *F* | *G* | *A* | *P* | *A* | *L* | *L* |
| H0XRB7 OTELMUR GARNETTI | P | A | . | A | G | L | . | S | S | V | V | P | G | T | A | L | Q | P | Q | E | S | V | **G** | A | G | G | S | . | . | . | . | . | E | A | A | L | . | P | L | S | G | L | L | *L* | *G* | *A* | *P* | *A* | *L* | *L* |
| G3U9C5 AFRICAN ELEPHANT | P | . | . | A | G | A | . | S | S | P | **A** | T | G | T | A | L | Q | P | Q | K | T | V | **G** | A | G | A | V | P | . | . | . | . | Q | G | A | L | . | P | L | P | G | L | L | *F* | *G* | *A* | *P* | *A* | *L* | *L* |
| H0W812 GUINEA PIG | . | . | . | . | . | . | . | . | S | Q | **A** | P | G | T | V | L | Q | P | Q | E | S | . | . | . | . | . | . | . | . | . | . | . | . | A | M | L | . | P | M | P | G | L | L | *F* | *G* | *A* | *P* | *V* | *L* | *L* |
| A0A6J2A960 CHEETAH | L | A | . | A | G | P | . | G | S | L | **A** | P | G | T | A | L | Q | P | Q | E | S | V | **G** | A | G | A | A | A | E | T | E | A | E | A | A | L | . | P | L | P | A | L | L | *F* | *G* | *A* | *P* | *A* | *L* | *L* |
| A0A6P6I3X8 PUMA | P | A | . | A | G | P | . | G | S | L | **A** | P | G | T | A | L | Q | P | Q | E | S | V | **G** | A | G | A | A | A | E | T | E | A | E | A | A | L | . | P | L | P | A | L | L | *F* | *G* | *A* | *P* | *A* | *L* | *L* |
| M3YY53 EUROPEAN POLECAT | P | A | . | A | G | A | S | S | S | P | **A** | P | G | T | A | L | Q | P | Q | E | S | V | **G** | P | G | G | A | A | . | . | E | A | E | A | A | L | . | P | L | P | A | L | L | *F* | *G* | *A* | *P* | *A* | *L* | *L* |
| A0A3Q7UGN9 GRIZZLY BEAR | P | A | . | A | G | A | . | S | S | L | **A** | P | G | T | A | L | Q | P | Q | E | S | V | **G** | P | G | T | A | A | . | . | E | A | E | A | A | L | . | P | L | P | A | L | L | *F* | *G* | *A* | *P* | *A* | *L* | *L* |
| A0A2U3ZCW5 WALRUS | P | . | . | A | R | A | . | S | S | L | **A** | P | G | T | A | L | Q | P | Q | E | S | V | **G** | P | G | A | A | A | . | . | E | A | D | T | A | L | . | P | L | P | A | L | L | *F* | *G* | *A* | *P* | *A* | *L* | *L* |
| A0A1S2ZRM0 EUROP. HEDGEHOG | P | . | . | A | G | T | . | S | S | L | E | P | G | T | A | L | Q | P | Q | E | S | V | **G** | S | G | V | A | P | . | . | . | A | E | G | A | L | . | P | L | P | A | L | L | *V* | *G* | *A* | *P* | *A* | *G* | *L* |
| F1MJD0 CATTLE | L | . | . | A | G | D | R | S | S | L | **A** | P | G | T | A | L | Q | P | Q | E | S | A | **G** | P | G | T | P | A | . | . | E | A | E | A | A | L | . | P | L | P | A | L | L | *F* | *G* | *A* | *P* | *A* | *L* | *L* |
| A0A452EY37 GOAT | L | A | . | G | G | P | . | S | S | L | **A** | P | G | T | A | L | Q | P | Q | E | S | A | **G** | P | G | T | P | A | . | . | E | A | E | A | A | L | . | P | L | P | A | L | L | *F* | *G* | *A* | *P* | *A* | *L* | *L* |
| A0A2Y9T0B2 SPERM WHALE | P | A | . | G | G | P | . | S | S | L | **A** | P | G | T | A | L | Q | P | Q | E | S | V | **G** | P | G | A | A | A | . | . | E | A | E | A | A | L | . | P | L | P | A | L | L | *F* | *G* | *A* | *P* | *A* | *L* | *L* |
| A0A6P3QWA8 FLYING FOX | P | . | . | A | G | P | . | S | S | L | **A** | P | G | T | A | L | Q | P | Q | E | S | V | **G** | A | G | T | P | A | . | . | H | A | E | A | A | L | . | P | L | P | A | L | L | *F* | *G* | *A* | *P* | *A* | *L* | *L* |
| A0A287AJ47 PIG | P | A | . | G | G | P | . | S | S | L | **A** | P | G | T | A | L | Q | P | Q | E | S | V | **G** | P | G | A | A | . | . | . | E | A | E | A | A | L | . | P | L | P | A | L | L | *L* | *G* | *A* | *P* | *A* | *L* | *L* |
| Q9D8D0 MOUSE | . | . | . | T | G | H | T | S | S | L | E | P | G | T | A | L | Q | P | Q | E | . | . | . | . | . | . | . | . | . | . | . | . | G | S | A | L | R | P | D | V | A | L | L | *V* | *G* | *A* | *P* | *A* | *L* | *L* |
| Q3BK46 CHICK | P | A | L | G | A | P | S | S | D | T | L | P | T | V | P | . | . | . | . | . | . | . | . | . | . | . | . | . | . | . | . | . | S | M | D | L | . | P | S | S | L | L | I | *F* | *G* | *V* | *P* | *V* | *L* | *V* |

|  |  |  |  | 9 | 0 |  | **92** | **-** | **-** | **-** | **95** |  |  | 1 | 0 | 0 |  |  |  |  |  |  |  |  |  |  | 1 | 1 | 0 |  |  |  |  |  |  |  |  |  |  | 1 | 2 | 0 |  |  |  |  |  | 1 | 2 | 5 |
| --- | --- | --- | --- | --- | --- | --- | --- | --- | --- | --- | --- | --- | --- | --- | --- | --- | --- | --- | --- | --- | --- | --- | --- | --- | --- | --- | --- | --- | --- | --- | --- | --- | --- | --- | --- | --- | --- | --- | --- | --- | --- | --- | --- | --- | --- | --- | --- | --- | --- | --- |
| Q96RJ3 HUMAN | *G* | *L* | *A* | *L* | *V* | *L* | ***A*** | ***L*** | ***V*** | *.* | ***L*** | *V* | *G* | *L* | *V* | S | W | R | R | . | . | . | R | Q | R | R | L | R | G | A | S | S | A | E | A | P | . | . | . | D | G | D | K | D | A | P | . | . | . | E |
| H2QLT1 CHIMPANZEE | *G* | *L* | *A* | *L* | *V* | *L* | ***A*** | ***L*** | ***V*** | *.* | ***L*** | *V* | *G* | *L* | *V* | S | W | R | R | . | . | . | R | Q | R | R | L | R | G | A | S | S | A | E | A | P | . | . | . | D | G | D | K | D | A | P | . | . | . | E |
| G3R8S3 GORILLA | *G* | *L* | *A* | *L* | *V* | *L* | ***A*** | ***L*** | ***V*** | *.* | ***L*** | *V* | *G* | *L* | *V* | S | W | R | R | . | . | . | R | Q | R | R | L | R | G | A | S | S | A | E | A | P | . | . | . | D | G | D | K | E | A | P | . | . | . | E |
| A0A5F7ZFG6 MACAQUE | *G* | *L* | *A* | *L* | *V* | *L* | ***A*** | ***L*** | ***V*** | *.* | ***L*** | *V* | *G* | *L* | *V* | S | W | R | R | . | . | . | R | Q | R | R | L | R | G | A | S | S | A | E | A | P | . | . | . | D | G | D | K | D | K | D | . | . | . | E |
| H9KVT2 MARMOSET | *G* | *L* | *V* | *L* | *V* | *L* | ***A*** | ***L*** | ***V*** | *.* | ***L*** | *V* | *G* | *L* | *V* | S | W | R | R | . | . | . | R | Q | Q | R | L | R | G | A | A | S | S | E | A | P | D | . | . | D | G | D | E | A | A | P | . | . | . | E |
| H0XRB7 OTELMUR GARNETTI | *G* | *L* | *A* | *L* | *A* | *L* | ***A*** | ***L*** | *A* | *.* | ***L*** | *V* | *G* | *L* | *V* | S | W | R | Y | . | . | . | . | . | R | R | L | R | G | A | A | S | P | Q | A | P | . | . | . | N | E | G | Q | H | . | . | . | . | . | E |
| G3U9C5 AFRICAN ELEPHANT | *G* | *L* | *A* | *.* | *.* | *L* | ***A*** | *M* | *G* | *.* | ***L*** | *V* | *R* | *L* | *V* | R | W | R | R | . | . | . | . | . | . | R | R | R | G | M | A | P | P | E | A | S | D | . | . | R | P | D | P | P | F | P | P | L | P | E |
| H0W812 GUINEA PIG | *G* | *L* | *A* | *L* | *L* | *L* | ***A*** | ***L*** | ***V*** | *.* | ***L*** | *V* | *A* | *L* | *V* | S | W | K | W | . | . | . | R | Q | R | R | T | Q | . | . | . | . | . | . | . | . | . | . | . | . | . | . | . | . | . | . | . | . | . | E |
| A0A6J2A960 CHEETAH | *G* | *L* | *A* | *.* | *.* | *L* | ***A*** | ***L*** | ***V*** | *.* | ***L*** | *V* | *G* | *L* | *L* | S | W | R | W | . | . | R | R | R | R | R | L | R | A | A | A | P | P | G | D | P | . | . | . | D | P | H | P | H | . | . | . | . | . | E |
| A0A6P6I3X8 PUMA | *G* | *L* | *A* | *.* | *.* | *L* | ***A*** | ***L*** | ***V*** | *.* | ***L*** | *V* | *G* | *L* | *L* | S | W | R | W | R | R | R | R | R | R | R | L | R | A | A | A | P | P | G | D | P | . | . | . | D | P | H | P | H | . | . | . | . | . | E |
| M3YY53 EUROPEAN POLECAT | *G* | *L* | *A* | *.* | *.* | *L* | ***A*** | ***L*** | ***V*** | *.* | ***L*** | *V* | *G* | *L* | *L* | G | C | R | W | . | . | . | R | R | R | R | L | R | A | G | A | P | P | G | T | P | . | . | . | E | P | Q | P | R | . | . | . | . | . | E |
| A0A3Q7UGN9 GRIZZLY BEAR | *G* | *L* | *A* | *.* | *.* | *L* | ***A*** | ***L*** | ***V*** | *.* | ***L*** | *V* | *G* | *L* | *L* | S | W | R | R | . | . | . | R | R | R | R | L | R | A | A | A | P | P | G | A | P | . | . | . | D | P | H | A | H | P | H | . | . | . | E |
| A0A2U3ZCW5 WALRUS | *G* | *L* | *A* | *.* | *.* | *L* | ***A*** | ***L*** | ***V*** | *.* | ***L*** | *V* | *G* | *L* | *L* | S | W | R | W | . | . | . | . | R | R | R | P | H | A | A | A | P | P | R | A | P | . | . | . | A | R | H | P | H | . | . | . | . | . | E |
| A0A1S2ZRM0 EUROP. HEDGEHOG | *G* | *L* | *A* | *.* | *.* | *L* | ***A*** | ***L*** | *L* | *.* | ***L*** | *L* | *G* | *L* | *L* | S | W | R | R | . | . | . | . | . | R | C | G | R | G | E | A | R | P | E | V | A | . | . | . | A | A | E | P | Q | E | . | . | . | . | E |
| F1MJD0 CATTLE | *G* | *L* | *A* | *.* | *.* | *L* | ***A*** | ***L*** | *A* | *.* | ***L*** | *L* | *G* | *L* | *V* | T | W | K | R | . | . | . | R | R | R | Q | P | G | A | A | V | V | P | E | A | P | . | . | . | E | A | A | E | V | P | . | . | . | . | E |
| A0A452EY37 GOAT | *G* | *L* | *A* | *.* | *.* | *L* | ***A*** | ***L*** | *A* | *.* | ***L*** | *I* | *G* | *L* | *V* | T | W | K | R | . | . | . | R | R | R | Q | P | G | A | A | V | V | P | E | A | P | . | . | . | . | . | . | . | . | . | . | . | . | . | E |
| A0A2Y9T0B2 SPERM WHALE | *G* | *L* | *A* | *.* | *.* | *L* | ***A*** | ***L*** | ***V*** | *.* | ***L*** | *L* | *G* | *L* | *V* | G | W | R | R | . | . | . | Q | R | R | R | P | G | A | A | A | A | P | E | A | L | E | A | R | D | G | D | Q | D | . | . | . | . | . | E |
| A0A6P3QWA8 FLYING FOX | *G* | *L* | *A* | *.* | *.* | *L* | ***A*** | ***L*** | ***V*** | *.* | ***L*** | *V* | *A* | *L* | *V* | S | W | R | Q | . | R | R | R | R | R | R | P | P | G | P | A | S | P | E | A | R | . | . | . | D | R | E | Q | D | . | . | . | . | . | E |
| A0A287AJ47 PIG | *G* | *L* | *A* | *.* | *.* | *L* | ***A*** | ***L*** | ***V*** | *.* | ***L*** | *L* | *G* | *L* | *V* | S | W | R | R | . | . | . | R | R | Q | R | P | R | A | S | A | A | P | E | A | P | E | A | L | D | G | D | Q | D | . | . | . | . | . | G |
| Q9D8D0 MOUSE | *G* | *L* | *I* | *L* | *A* | *L* | *T* | *L* | *V* | *G* | ***L*** | *V* | *S* | *L* | *V* | S | W | R | W | . | . | . | . | R | Q | Q | L | R | . | T | A | S | P | D | T | S | . | . | . | E | G | V | Q | Q | . | . | . | . | . | E |
| Q3BK46 CHICK | *G* | *L* | *L* | *.* | *.* | *L* | ***A*** | ***L*** | *A* | *A* | ***L*** | *W* | *G* | *F* | *L* | A | C | K | L | G | K | R | R | R | K | R | R | K | A | E | Q | E | A | E | E | S | . | . | . | H | G | D | A | . | . | . | . | . | . | G |

|  |  |  | 1 | 3 | 0 |  |  |  |  |  |  |  | 1 | 4 | 0 |  |  |  |  |  |  |  |  |  |  |  |  |  |  |  |  |  |  |  |  |  |  |  |  |  |  |  |  |  |  | 1 | 4 | 6 |  |  |
| --- | --- | --- | --- | --- | --- | --- | --- | --- | --- | --- | --- | --- | --- | --- | --- | --- | --- | --- | --- | --- | --- | --- | --- | --- | --- | --- | --- | --- | --- | --- | --- | --- | --- | --- | --- | --- | --- | --- | --- | --- | --- | --- | --- | --- | --- | --- | --- | --- | --- | --- |
| Q96RJ3 HUMAN | P | L | D | K | V | I | I | L | S | P | G | I | S | D | A | T | A | P | . | . | . | . | . | . | . | . | . | . | . | . | . | . | . | . | . | . | . | . | . | . | . | . | . | . | . | A | W | **P** | P | P |
| H2QLT1 CHIMPANZEE | P | L | D | K | V | I | I | L | S | P | A | I | S | D | A | T | A | P | . | . | . | . | . | . | . | . | . | . | . | . | . | . | . | . | . | . | . | . | . | . | . | . | . | . | . | A | W | **P** | P | P |
| G3R8S3 GORILLA | P | L | D | K | V | I | I | L | S | P | G | I | S | D | A | T | A | P | . | . | . | . | . | . | . | . | . | . | . | . | . | . | . | . | . | . | . | . | . | . | . | . | . | . | . | A | W | **P** | P | P |
| A0A5F7ZFG6 MACAQUE | P | L | D | K | V | I | I | L | S | P | G | I | S | D | A | A | A | P | . | . | . | . | . | . | . | . | . | . | . | . | . | . | . | . | . | . | . | . | . | . | . | . | . | . | . | A | W | **P** | P | P |
| H9KVT2 MARMOSET | P | L | D | K | L | I | I | L | S | P | G | T | T | D | A | T | A | S | . | . | . | . | . | . | . | . | . | . | . | . | . | . | . | . | . | . | . | . | . | . | . | . | . | . | . | A | W | **P** | P | P |
| H0XRB7 OTELMUR GARNETTI | P | L | D | N | V | I | V | L | S | P | G | T | I | D | T | T | A | P | . | . | . | . | . | . | . | . | . | . | . | . | . | . | . | . | . | . | . | . | . | . | . | . | . | . | . | V | W | **P** | L | P |
| G3U9C5 AFRICAN ELEPHANT | S | L | D | N | V | I | I | L | S | P | G | P | I | D | A | T | S | P | . | . | . | . | . | . | . | . | . | . | . | . | . | . | . | . | . | . | . | . | . | . | . | . | . | . | . | V | W | S | P | P |
| H0W812 GUINEA PIG | D | L | S | C | V | Y | I | L | S | S | G | T | P | D | A | S | A | P | . | . | . | . | . | . | . | . | . | . | . | . | . | . | . | . | . | . | . | . | . | . | . | . | . | . | . | D | W | **P** | T | P |
| A0A6J2A960 CHEETAH | P | L | D | D | V | I | I | H | P | P | K | P | L | D | A | T | A | P | . | . | . | . | . | . | . | . | . | . | . | . | . | . | . | . | . | . | . | . | . | . | . | . | . | . | . | V | W | **P** | P | P |
| A0A6P6I3X8 PUMA | P | L | D | D | V | I | I | H | P | P | K | P | L | D | A | T | A | P | . | . | . | . | . | . | . | . | . | . | . | . | . | . | . | . | . | . | . | . | . | . | . | . | . | . | . | V | W | **P** | P | P |
| M3YY53 EUROPEAN POLECAT | T | L | D | H | V | T | I | L | P | P | G | P | L | D | A | T | A | P | . | . | . | . | . | . | . | . | . | . | . | . | . | . | . | . | . | . | . | . | . | . | . | . | . | . | . | I | W | **P** | P | T |
| A0A3Q7UGN9 GRIZZLY BEAR | P | L | D | N | V | I | V | L | P | P | G | P | L | D | A | T | A | P | . | . | . | . | . | . | . | . | . | . | . | . | . | . | . | . | . | . | . | . | . | . | . | . | . | . | . | V | W | **P** | P | T |
| A0A2U3ZCW5 WALRUS | A | L | D | N | V | I | I | L | P | P | G | P | L | D | A | T | A | P | . | . | . | . | . | . | . | . | . | . | . | . | . | . | . | . | . | . | . | . | . | . | . | . | . | . | . | V | W | **P** | P | T |
| A0A1S2ZRM0 EUROP. HEDGEHOG | P | L | D | H | V | I | I | L | P | P | E | P | L | L | A | T | A | P | . | . | . | . | . | . | . | . | . | . | . | . | . | . | . | . | . | . | . | . | . | . | . | . | . | . | . | V | W | **P** | P | P |
| F1MJD0 CATTLE | S | L | D | V | D | T | T | L | S | P | G | T | L | D | A | T | A | P | . | . | . | . | . | . | . | . | . | . | . | . | . | . | . | . | . | . | . | . | . | . | . | . | . | . | . | I | W | L | L | P |
| A0A452EY37 GOAT | P | L | D | V | D | T | T | . | . | P | G | T | L | D | A | T | A | P | . | . | . | . | . | . | . | . | . | . | . | . | . | . | . | . | . | . | . | . | . | . | . | . | . | . | . | I | W | L | L | P |
| A0A2Y9T0B2 SPERM WHALE | P | L | D | S | D | N | I | L | S | P | G | T | L | D | A | T | A | P | . | . | . | . | . | . | . | . | . | . | . | . | . | . | . | . | . | . | . | . | . | . | . | . | . | . | . | I | W | L | P | P |
| A0A6P3QWA8 FLYING FOX | P | L | N | S | V | . | I | L | S | P | G | P | L | D | A | T | A | P | . | . | . | . | . | . | . | . | . | . | . | . | . | . | . | . | . | . | . | . | . | . | . | . | . | . | . | I | W | **P** | P | P |
| A0A287AJ47 PIG | P | L | D | N | D | I | V | P | S | P | G | T | L | D | A | T | A | P | . | . | . | . | . | . | . | . | . | . | . | . | . | . | . | . | . | . | . | . | . | . | . | . | . | . | . | I | W | L | P | P |
| Q9D8D0 MOUSE | S | L | E | N | V | F | V | P | S | S | E | T | P | H | A | S | A | P | . | . | . | . | . | . | . | . | . | . | . | . | . | . | . | . | . | . | . | . | . | . | . | . | . | . | . | T | W | **P** | P | L |
| Q3BK46 CHICK | P | L | P | S | . | . | . | . | . | S | G | C | L | D | V | S | T | P | E | G | S | A | D | P | A | Q | G | H | C | P | H | R | N | G | G | M | R | M | P | R | R | D | G | A | K | Q | W | **P** | C | C |

|  | 1 | 5 | 0 |  |  |  |  |  |  |  |  |  |  |  |  |  |  |  |  |  | 1 | 5 | 9 |  |  |  |  |  |  |  |  | 1 | 7 | 0 |  |  |  |  |  |  |  |  |  |  |  |  |  |  |  | 1 | 8 | 4 |
| --- | --- | --- | --- | --- | --- | --- | --- | --- | --- | --- | --- | --- | --- | --- | --- | --- | --- | --- | --- | --- | --- | --- | --- | --- | --- | --- | --- | --- | --- | --- | --- | --- | --- | --- | --- | --- | --- | --- | --- | --- | --- | --- | --- | --- | --- | --- | --- | --- | --- | --- | --- | --- |
| Q96RJ3 HUMAN | . | G | E | D | P | G | . | . | . | . | . | . | T | T | P | P | . | . | . | . | . | G | **H** | **S** | **V** | **P** | **V** | **P** | **A** | **T** | **E** | **L** | **G** | **S** | **T** | E | L | V | T | T | K | T | A | . | . | . | . | G | P | E | Q | Q |
| H2QLT1 CHIMPANZEE | . | G | E | D | P | G | . | . | . | . | . | . | T | T | P | P | . | . | . | . | . | G | **H** | **S** | **V** | **P** | **V** | **P** | **A** | **T** | **E** | **L** | **G** | **S** | **T** | E | L | V | T | T | K | T | A | . | . | . | . | G | P | E | Q | Q |
| G3R8S3 GORILLA | . | G | E | D | P | G | . | . | . | . | . | . | T | T | P | P | . | . | . | . | . | G | **H** | **S** | **V** | **P** | **V** | **P** | **A** | **T** | **E** | **L** | **G** | **S** | **T** | E | L | V | T | T | K | T | A | . | . | . | . | G | P | E | Q | Q |
| A0A5F7ZFG6 MACAQUE | . | G | E | D | P | G | . | . | . | . | . | . | T | T | P | P | . | . | . | . | . | G | **H** | **S** | **V** | **P** | **V** | **P** | **A** | **T** | **E** | **L** | **G** | **S** | **T** | E | L | V | T | T | K | T | A | . | . | . | . | G | P | E | Q | Q |
| H9KVT2 MARMOSET | . | G | E | D | P | G | . | . | . | . | . | . | T | T | P | P | . | . | . | . | . | G | **H** | **S** | **V** | **P** | **V** | **P** | **A** | **T** | **E** | **L** | **G** | **S** | **T** | E | L | V | T | T | K | T | A | . | . | . | . | G | P | E | Q |  |
| H0XRB7 OTELMUR GARNETTI | R | E | E | D | P | G | . | . | . | . | . | . | T | T | P | P | . | . | . | . | . | G | **H** | **S** | **V** | **P** | **V** | **P** | **A** | **T** | **E** | **L** | **G** | **S** | **T** | E | L | V | T | T | K | T | A | . | . | . | . | G | L | E | Q |  |
| G3U9C5 AFRICAN ELEPHANT | . | . | E | D | R | A | . | . | . | . | . | . | T | T | L | P | . | . | . | . | . | G | **H** | **S** | **V** | **P** | **V** | **P** | **A** | **T** | **E** | **L** | **G** | **S** | **T** | E | L | V | T | T | K | T | T | . | . | . | . | G | P | E | Q |  |
| H0W812 GUINEA PIG | . | R | E | D | P | A | . | . | . | . | . | . | T | T | P | S | . | . | . | . | . | S | **H** | **S** | **V** | **P** | **V** | **P** | **A** | **T** | **E** | **L** | **G** | **S** | **T** | E | L | V | T | T | K | T | A | . | . | . | . | G | P | E | Q |  |
| A0A6J2A960 CHEETAH | . | R | E | D | P | A | . | . | . | . | . | . | N | T | P | P | . | . | . | . | . | A | **H** | **S** | **V** | **P** | **V** | **P** | **A** | **T** | **E** | **L** | **G** | **S** | **T** | E | L | V | T | T | K | T | A | . | . | . | . | G | P | E | Q | Q |
| A0A6P6I3X8 PUMA | . | R | E | D | P | A | . | . | . | . | . | . | N | T | P | P | . | . | . | . | . | A | **H** | **S** | **V** | **P** | **V** | **P** | **A** | **T** | **E** | **L** | **G** | **S** | **T** | E | L | V | T | T | K | T | A | . | . | . | . | G | P | E | Q | Q |
| M3YY53 EUROPEAN POLECAT | . | R | E | D | T | A | . | . | . | . | . | . | N | T | P | P | . | . | . | . | . | A | **H** | **S** | **V** | **P** | **V** | **P** | **A** | **T** | **E** | **L** | **G** | **S** | **T** | E | L | V | T | T | K | T | A | . | . | . | . | G | P | E | Q |  |
| A0A3Q7UGN9 GRIZZLY BEAR | . | G | E | D | P | A | . | . | . | . | . | . | N | T | P | P | . | . | . | . | . | A | **H** | **S** | **V** | **P** | **V** | **P** | **A** | **T** | **E** | **L** | **G** | **S** | **T** | E | L | V | T | T | K | T | A | . | . | . | . | G | P | E | Q | Q |
| A0A2U3ZCW5 WALRUS | . | R | E | D | P | A | . | . | . | . | . | . | N | T | P | P | . | . | . | . | . | A | **H** | **S** | **V** | **P** | **V** | **P** | **A** | **T** | **E** | **L** | **G** | **S** | **T** | E | L | V | T | T | K | T | A | . | . | . | . | G | P | E | Q | Q |
| A0A1S2ZRM0 EUROP. HEDGEHOG | . | R | D | D | P | G | . | . | . | . | . | . | A | T | P | T | . | . | . | . | . | A | **H** | **S** | **V** | **P** | **V** | **P** | **A** | **T** | **E** | **L** | **G** | **S** | **T** | E | L | V | T | T | K | T | A | . | . | . | . | G | P | E | Q | Q |
| F1MJD0 CATTLE | . | S | E | D | P | A | . | . | . | . | . | . | A | T | P | P | . | . | . | . | . | T | **H** | **S** | **V** | **P** | **V** | **P** | **A** | **T** | **E** | **L** | **G** | **S** | **T** | E | L | V | T | T | K | T | A | . | . | . | . | G | P | E | L | Q |
| A0A452EY37 GOAT | . | S | E | D | P | A | . | . | . | . | . | . | A | T | P | P | . | . | . | . | . | T | **H** | **S** | **V** | **P** | **V** | **P** | **A** | **T** | **E** | **L** | **G** | **S** | **T** | E | L | V | T | T | K | T | T | . | . | . | . | G | P | E | L | Q |
| A0A2Y9T0B2 SPERM WHALE | . | S | E | D | P | A | . | . | . | . | . | . | T | T | P | P | . | . | . | . | . | A | **H** | **S** | **V** | **P** | **V** | **P** | **A** | **T** | **E** | **L** | **G** | **S** | **T** | E | L | V | T | T | K | T | A | . | . | . | . | G | P | E | Q | P |
| A0A6P3QWA8 FLYING FOX | . | T | E | D | S | A | . | . | . | . | . | . | T | T | P | P | . | . | . | . | . | A | **H** | **S** | **V** | **P** | **V** | **P** | **A** | **T** | **E** | **L** | **G** | **S** | **T** | E | L | V | T | T | K | T | A | . | . | . | . | G | P | E | Q | Q |
| A0A287AJ47 PIG | . | S | E | D | S | A | . | . | . | . | . | . | T | T | P | P | . | . | . | . | . | A | **H** | **S** | **V** | **P** | **V** | **P** | **A** | **T** | **E** | **L** | **G** | **S** | **T** | E | L | V | T | T | K | T | A | . | . | . | . | G | P | E | Q | Q |
| Q9D8D0 MOUSE | . | K | E | D | A | D | . | . | . | . | . | . | S | A | L | P | . | . | . | . | . | R | **H** | **S** | **V** | **P** | **V** | **P** | **A** | **T** | **E** | **L** | **G** | **S** | **T** | E | L | V | T | T | K | T | A | . | . | . | . | G | P | E | Q |  |
| Q3BK46 CHICK | Q | G | D | A | K | G | D | V | V | L | L | A | T | T | W | P | H | H | K | E | H | G | **H** | **S** | F | **P** | L | **P** | **A** | **T** | **E** | **L** | **G** | A | **T** | A | L | V | T | T | K | T | T | Q | E | C | M | G | E | E | R | L |

Amino acid residues changed by the variants are shown in bold red letters. The cysteine residues of the CRD in blue and bold, the residues in the transmembrane region in italics, and the TRAF3 binding region in bold green and letters. The numbering refers to the human amino acid sequence of BAFFR.

# Supplemental Figures

| **** |
| --- |

# Supplemental Figure S1. Analysis of PBMCs from CVID patients carrying different BAFFR variants.

**a.** **B cell subsets**. The FACS profiles show B cell subsets of heterozyogous P21R, A52T, G64V, DUP92-95, of a homozygous H159Y carrier, and of a healthy control. CD19-positive B cells were gated into naïve (IgD+ CD27-), marginal zone (IgD+ CD27+) and memory B cells (IgD- CD27+).

**b. BAFFR expression levels.** Histogram overlays of BAFFR surface levels detected by mAb HuBR9.1 (for P21R) or mAB 11C1 (for all other variants) on CD19+ IgD+ CD27- B cells of CVID patients with different BAFFR variants (red histogram) and matched controls (black histogram). Since the BAFFR-A52T carrier was analyzed 6 month after treatment with rituximab, expression was also compared to cord blood cells as control.

|  |
| --- |
| Supplemental Figure S2. Expression of WT and variant BAFF-GFP fusion proteins by Lentivirally transduced DG-75 BAFFR KO cells. **a.** **BAFFR surface expression detected by mAb11C1.** Cell lines transduced with BAFFR-fusion proteins were stained with APC-conjugated 11C1 and analyzed by flow cytometry for BAFFR expression lon the surface of DG-75 cells. The histogram overlays show that the P21R variant does not bind 11C1.  **b.** **Histogram overlays of 4 of DG-75 BAFFR KO cells expressing WT or variant BAFFR-GFP fusion proteins.** The overlays show 4 independent series of experiments with cells analyzed by flow cytometry using the anti-human BAFFR mAb HuBR9.1. Different from 11C1, the antibody binds to P21R. **c. Histogram overlays of GFP expressed from the BAFFR fusion proteins displayed in (b).** **d.** **Quantification of the peak fluorescence (mode) of BAFFR (HuBR9.1) and of the GFP signals** (**e**) of the the histograms shown in panels (b) and (c). **f.** **Differences in the expression of BAFFR between DG-75 BAFFR KO cells expressing WT or variant BAFFR** as detected by mAb HuBR9.1. The differences in expression levels were calculated by the ratio of the signals of variant BAFFR/WT BAFFR obtained from the analysis shown in (d). **g.** **Differences in BAFFR expression by the different cell lines calculated by the ratio of GFP signals of variant /WT BAFFR fusion proteins.** The signal intensities were obtained from the analysis shown in (e). **h. Ratio of BAFFR (HuBR9.1)/GFP signals for each cell line expressing WT or variant BAFFR fusion proteins.** Data were obtained from the signal intensities determined in (d,e). Statisitically significant differences were calculated by Brown-Forsythe and Welch's ANOVA for multiple comparisions with Dunn's T3 correction for n<50.  The histogram overlays and the comparison of HuBR9.1 and GFP signal intensities show that the individual spread between different experiments with the same cell lines is larger for HuBR9.1 than for GFP. Therefore, GFP signals were used to account for the differences in the expression of BAFFR-GFP fusion proteins by the DG-75 cell lines expressing WT or variant BAFFRs. |

| **** |
| --- |

# Supplemental Fig. S3 Gating strategy for flow-based FRET analysis and BAFF-induced changes of FRET signals.

**a.** DG-75 BAFFR KO cells were transduced with lentiviral vectors encoding WT BAFFR-GFP and WT BAFFR-RFP and analyzed by flow cytometry using a LSR Fortessa II cytometer and FloJo software. The cells were plotted based on size and granularity and the B cell population was gated before and 10 min after addition of BAFF.

**b.** The lentiviral transduction led to four different populations (GFP+, RFP-; GFP+, RFP+; GFP-, RFP- cells). FRET signals excited by the 488 nm laser resulting from energy transfer from GFP to RFP were detected in form of RFP fluorescence with a 625/15 nm bandpass filter . After the appropriate compensation of the spillover signals from GFP and RFP emission into the channel detecting FRET signals, the gates for FRET+ cells were set using the single-positive GFP+ RFP- and GFP-RFP+ populations. The plots display the FRET signals before adding BAFF.

**c.** **Gating strategy of FRET analysis.** As described in (b) DG-75 cells transduced with GFP and RFP were analyzed 10 min after adding 50ng/ml BAFF.

**d.** **Increase of FRET signals induced by BAFF binding to BAFFR**. FRET signals from DG-75 cells expressing BAFFR-GFP and BAFFR-RFP were continuously recorded by flow cytometry as described in (b-c). After 300 seconds (5 min), 50 ng/ml of BAFF were added and the recording was continued for another 10 min (900 sec timepoint).

|  |
| --- |

# Supplemental Figure. S4. Co-immunoprecipitation of TRAF3 with FLAG-tagged BAFF bound to BAFFR.

DG-75 BAFFR KO cells expressing WT or variant BAFFRs from integrated lentiviral vectors carrying also IRES-GFP were stimulated in time course experiments with FLAG-tagged BAFF (0, 15 and 60min) and cell lysates were incubated with anti-FLAG coupled magnetic beads for 4h. TRAF3 and BAFFR co-immunoprecipitating with FLAG-BAFF were eluted from anti-FLAG IgG beads and detected by western blotting. The G64V mutation results in two BAFFR-specific signals.

| **** |
| --- |

# Supplemental Figure S5. Gating strategy to analyzed FRET between BAFFR and CD79B; analysis of CD79AKO and CD79B KO DG-75 cells; localization and potential structure of DUP92-95.

**a. Gating strategy for FRET analysis.** DG-75 BAFFR KO cells were transduced with lentiviral vectors encoding for BAFFR-GFP and CD79B-RFP resulting in four populations (GFP+,RFP-; GFP-, RFP+; GFP+,RFP+; GFP-, RFP-). To detect FRET induced by energy transfer from GFP to RFP, the cells were gated first according to size (FSC-A), granularity (SSC-A), and aggregation (FSC-A/ FSC-H). FRET signals generated by the energy transfer from the GFP donor fluorochrome (excited by the 488 nm laser) to the RFP acceptor fluorophore were detected by RFP fluorescence applying a 625/15 nm bandpass filter. After compensating spillover signals from GFP and RFP emission into the channel detecting FRET signals, the gates for FRET+ cells were set using the single-positive GFP+ RFP- and GFP-RFP+ populations. The plots display the FRET signals before adding BAFF.

**b.** **Inactivation of CD79A and CD79B in DG-75 cells.** *CD79A* and *CD79B* were inactivated in DG-75 cells by CRISPR/Cas9 mutagenesis. The inactivation was confirmed by flow cytometry analyzing the intracellular (CD79A) and the cell surface expression of CD79B. Since CD79B surface expression requires CD79A, CD79A KO cells do not express CD79B on the cell surface. Inactivation of one of the BCR signaling subunits did not change BAFFR surface expression levels as shown by the left histogram.

**c.** **Potential leucine zippers in the BAFFR TM region.** To potential leucine zippers (L77, L84, L91, L98) and L78, L85 are aligned to the leucine zipper motif. The 92-95 duplication shortens the longer leucine zipper by one repeat.

**d.** **3D models of the transmembrane regions of dimeric WT BAFFR (left) and DUP92-95 (right).** The transmembrane regions between P82 and V99 are shown in blue, the residues A92 - L95 in red.

| **** | Supplemental Figure S6. Western blot analysis of AKT S473 phosphorylation in DG-75 cell lines. 1 x 10^6^ DG75 BAFFR KO cells expressing WT or variant BAFFR from integrated lentiviral vectors carrying also IRES-GFP were treated with 100 ng/ml BAFF for the indicated time periods (0, 2, 5, 10, 30, 60 min). Whole cell lysates were analyzed by immunoblotting for AKT phosphorylation at the S473 residue. Beta actin was used as control to account for differences in cell numbers and protein concentrations between different samples- PS = protein standard. |
| --- | --- |

| **** |
| --- |

# Supplemental Figure S7. Phosphorylation of the small ribosomal protein S6 in naïve IgD+ CD27- B cells from CVID patients carrying the different variants.

Cells were stained first for the expression of CD19, IgD and CD27 on the cell surface followed by intracellular staining for pS6. The graph shows the percentages of pS6+ cells in the CD19+ IgD+ CD27- population representing naive B cells of individuals with the WT BAFFR or (a) P21R, (b) G64V, (c) DUP92-95, (d) H159Y variants. PBMC were activated for 1h with 20 ng/ml BAFF or with CD40L as described (11, 12). The population was chosen as it showed the smallest difference between all individuals tested. Each data point represents an independently analyzed replicate.

| **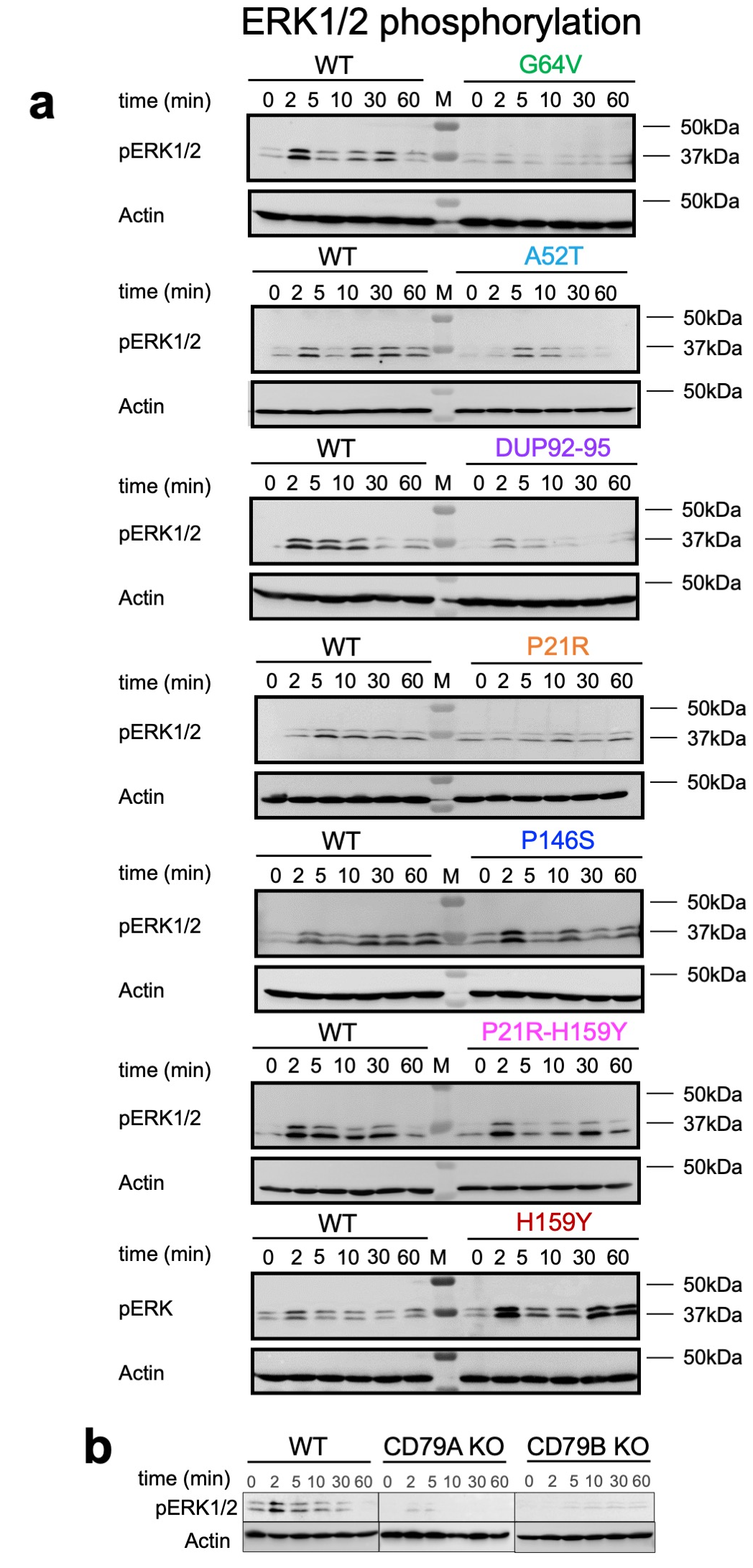** | Supplemental Figure S8. Western blot analysis of ERK 1/2 phosphorylation in DG-75 cell lines. **a.** 1. x 10^6^ DG-75 BAFFR KO cells expressing WT or variant BAFFR were treated with 100 ng/ml BAFF in a time dependent manner (0, 2, 5, 10, 30, 60 min) and analyzed by immunoblotting for pERK1/2 T202/Y204 and actin as loading control.  **b.** CD79A KO, CD79B KO or WT DG-75 cells expressing WT BAFFR-IRES-GFP were treated with 100 ng/ml BAFF in a time dependent manner (0-60 min) and analyzed western blot for pERK1/2 T202/Y204 and actin as loading control. |
| --- | --- |

|  |
| --- |

**
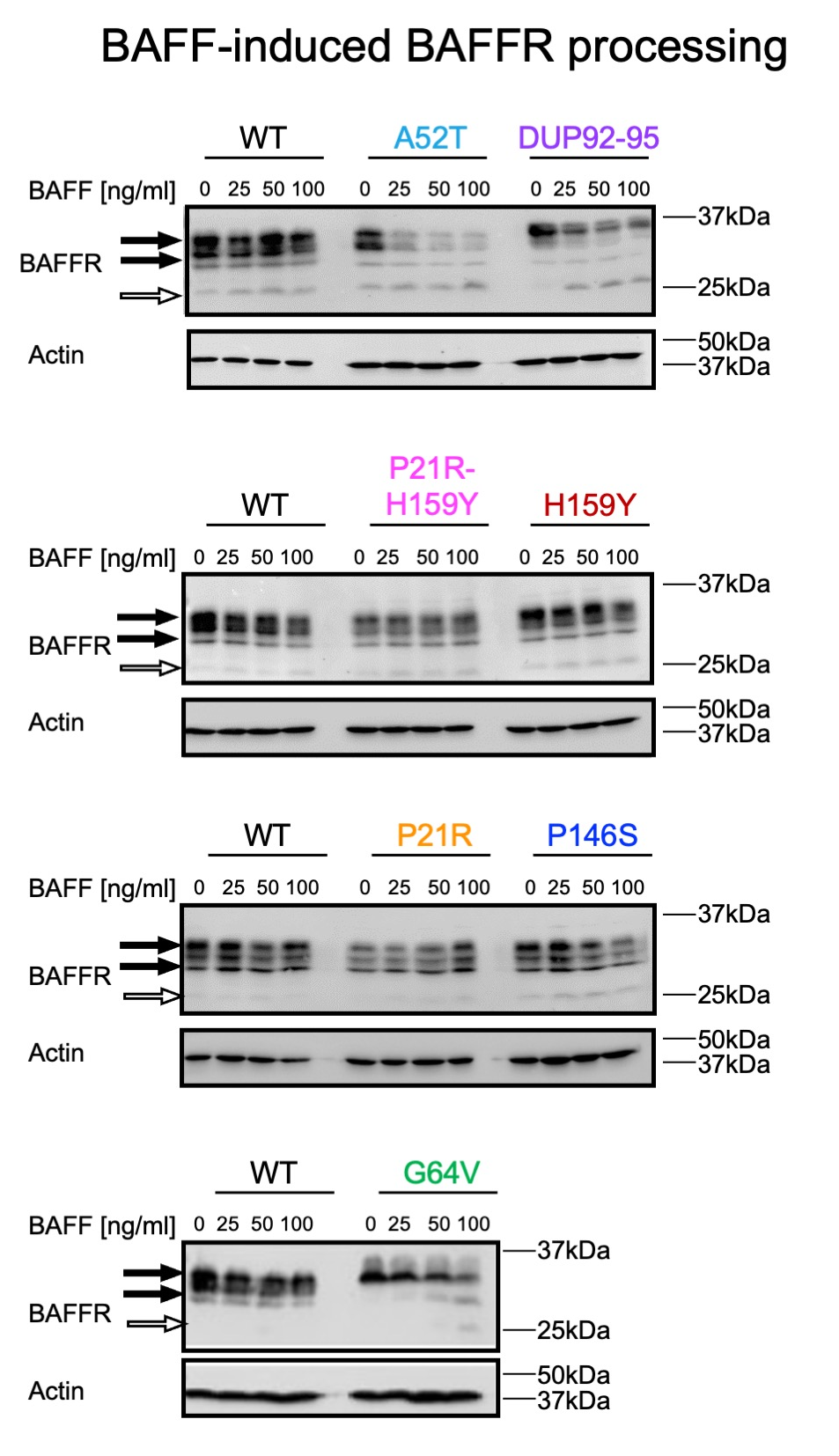
**

# Supplemental Fig. S9 BAFF induced BAFFR processing

BAFFR protein levels in whole cell lysates of DG-75 BAFFR KO cells transduced with WT BAFFR or the different BAFFR variants from integrated lentiviral vectors carrying also IRES-GFP were analyzed by western blot after an overnight dose response treatment with BAFF (0, 25, 50 and 100 ng/ml). Actin expression was used as loading control to account for differences in protein concentrations between different samples. Solid arrows show full-length BAFFR, open arrows the C-terminal fragment created by processing of the BAFFR ectodomain.

# References

1. Lougaris V, Baronio M, Castagna A, Tessarin G, Rossi S, Gazzurelli L, et al. Paediatric MAS/HLH caused by a novel monoallelic activating mutation in p110δ. Clin Immunol. 2020;219:108543.

2. Abolhassani H, Aghamohammadi A, Fang M, Rezaei N, Jiang C, Liu X, et al. Clinical implications of systematic phenotyping and exome sequencing in patients with primary antibody deficiency. Genet Med. 2019;21(1):243-51.

3. Abolhassani H, Hammarström L, Cunningham-Rundles C. Current genetic landscape in common variable immune deficiency. Blood. 2020;135(9):656-67.

4. Rojas-Restrepo J, Caballero-Oteyza A, Huebscher K, Haberstroh H, Fliegauf M, Keller B, et al. Establishing the Molecular Diagnoses in a Cohort of 291 Patients With Predominantly Antibody Deficiency by Targeted Next-Generation Sequencing: Experience From a Monocentric Study. Front Immunol. 2021;12:786516.

5. Fang M, Su Z, Abolhassani H, Itan Y, Jin X, Hammarstrom L. VIPPID: a gene-specific single nucleotide variant pathogenicity prediction tool for primary immunodeficiency diseases. Brief Bioinform. 2022.

6. Fang M, Abolhassani H, Lim CK, Zhang J, Hammarstrom L. Next Generation Sequencing Data Analysis in Primary Immunodeficiency Disorders - Future Directions. J Clin Immunol. 2016;36 Suppl 1:68-75.

7. Richards S, Aziz N, Bale S, Bick D, Das S, Gastier-Foster J, et al. Standards and guidelines for the interpretation of sequence variants: a joint consensus recommendation of the American College of Medical Genetics and Genomics and the Association for Molecular Pathology. Genet Med. 2015;17(5):405-24.

8. Burgener AV, Bantug GR, Meyer BJ, Higgins R, Ghosh A, Bignucolo O, et al. SDHA gain-of-function engages inflammatory mitochondrial retrograde signaling via KEAP1-Nrf2. Nat Immunol. 2019;20(10):1311-21.

9. Pieper K, Rizzi M, Speletas M, Smulski CR, Sic H, Kraus H, et al. A common single nucleotide polymorphism impairs B-cell activating factor receptor's multimerization, contributing to common variable immunodeficiency. The Journal of allergy and clinical immunology. 2014;133(4):1222-5.

10. Ntellas P, Dardiotis E, Sevdali E, Siokas V, Aloizou AM, Tsinti G, et al. TNFRSF13C/BAFFR P21R and H159Y polymorphisms in multiple sclerosis. Mult Scler Relat Disord. 2020;37:101422.

11. Smulski CR, Kury P, Seidel LM, Staiger HS, Edinger AK, Willen L, et al. BAFF- and TACI-Dependent Processing of BAFFR by ADAM Proteases Regulates the Survival of B Cells. Cell reports. 2017;18(9):2189-202.

12. Smulski CR, Zhang L, Burek M, Teixido Rubio A, Briem JS, Sica MP, et al. Ligand-independent oligomerization of TACI is controlled by the transmembrane domain and regulates proliferation of activated B cells. Cell reports. 2022;38(13):110583.

13. Schneider P, Willen L, Smulski CR. Tools and techniques to study ligand-receptor interactions and receptor activation by TNF superfamily members. Methods Enzymol. 2014;545:103-25.

14. Eswar N, Webb B, Marti-Renom MA, Madhusudhan MS, Eramian D, Shen MY, et al. Comparative protein structure modeling using Modeller. (1934-340X (Electronic)).

15. Pettersen EF, Goddard TD, Huang CC, Couch GS, Greenblatt DM, Meng EC, et al. UCSF Chimera--a visualization system for exploratory research and analysis. J Comput Chem. 2004;25(13):1605-12.

16. Pettersen EF, Goddard TD, Huang CC, Meng EC, Couch GS, Croll TI, et al. UCSF ChimeraX: Structure visualization for researchers, educators, and developers. (1469-896X (Electronic)).

17. Yang Z, Lasker K, Schneidman-Duhovny D, Webb B, Huang CC, Pettersen EF, et al. UCSF Chimera, MODELLER, and IMP: an integrated modeling system. J Struct Biol. 2012;179(3):269-78.

18. Waterhouse A, Bertoni M, Bienert S, Studer G, Tauriello G, Gumienny R, et al. SWISS-MODEL: homology modelling of protein structures and complexes. Nucleic Acids Research. 2018;46(W1):W296-W303.

19. Jumper J, Evans R, Pritzel A, Green T, Figurnov M, Ronneberger O, et al. Highly accurate protein structure prediction with AlphaFold. Nature. 2021;596(7873):583-9.
